# Less is more: low expression of MT1-MMP is optimal to promote migration and tumourigenesis of breast cancer cells

**DOI:** 10.1186/s12943-016-0547-x

**Published:** 2016-10-18

**Authors:** Mario A. Cepeda, Jacob J. H. Pelling, Caitlin L. Evered, Karla C. Williams, Zoey Freedman, Ioana Stan, Jessica A. Willson, Hon S. Leong, Sashko Damjanovski

**Affiliations:** 1 Department of Biology, Faculty of Science, University of Western Ontario, 1151 Richmond St N., London, Ontario N6A 5B7 Canada; 2 Department of Surgery, Schulich School of Medicine and Dentistry, University of Western Ontario, London, ON Canada; 3 Translational Prostate Cancer Research Laboratory, Lawson Health Research Institute, London, ON Canada; 4 Lawson Health Research Institute, London, ON Canada

**Keywords:** MT1-MMP, MMP-14, Cell migration, 3D culture, Breast cancer, Intravital imaging

## Abstract

**Background:**

Membrane Type-1 Matrix Metalloproteinase (MT1-MMP) is a multifunctional protease implicated in metastatic progression ostensibly due to its ability to degrade extracellular matrix (ECM) components and allow migration of cells through the basement membrane. Despite in vitro studies demonstrating this principle, this knowledge has not translated into the use of MMP inhibitors (MMPi) as effective cancer therapeutics, or been corroborated by evidence of in vivo ECM degradation mediated by MT1-MMP, suggesting that our understanding of the role of MT1-MMP in cancer progression is incomplete.

**Methods:**

MCF-7 and MDA-MB 231 breast cancer cell lines were created that stably overexpress different levels of MT1-MMP. Using 2D culture, we analyzed proMMP-2 activation (gelatin zymography), ECM degradation (fluorescent gelatin), ERK signaling (immunoblot), cell migration (transwell/scratch closure/time-lapse imaging), and viability (colorimetric substrate) to assess how different MT1-MMP levels affect these cellular parameters. We also utilized Matrigel 3D cell culture and avian embryos to examine how different levels of MT1-MMP expression affect morphological changes in 3D culture, and tumourigenecity and extravasation efficiency in vivo*.*

**Results:**

In 2D culture, breast cancer cells expressing high levels of MT1-MMP were capable of widespread ECM degradation and TIMP-2-mediated proMMP-2 activation, but were not the most migratory. Instead, cells expressing low levels of MT1-MMP were the most migratory, and demonstrated increased viability and ERK activation. In 3D culture, MCF-7 breast cancer cells expressing low levels of MT1-MMP demonstrated an invasive protrusive phenotype, whereas cells expressing high levels of MT1-MMP demonstrated loss of colony structure and cell fragment release. Similarly, in vivo analysis demonstrated increased tumourigenecity and metastatic capability for cells expressing low levels of MT1-MMP*,* whereas cells expressing high levels were devoid of these qualities despite the production of functional MT1-MMP protein.

**Conclusions:**

This study demonstrates that excessive ECM degradation mediated by high levels of MT1-MMP is not associated with cell migration and tumourigenesis, while low levels of MT1-MMP promote invasion and vascularization in vivo.

**Electronic supplementary material:**

The online version of this article (doi:10.1186/s12943-016-0547-x) contains supplementary material, which is available to authorized users.

## Background

During metastatic progression, cancer cells detach from the primary tumour into the surrounding extracellular matrix (ECM). A subpopulation of these cells will intravasate into the blood stream or lymphatic vessels, entering the circulation [1]. Due to their size, these disseminated cancer cells will eventually extravasate via transendothelial migration, and a small proportion will go onto proliferate to form secondary colonies [2–4]. Matrix Metalloproteinases (MMPs) have been implicated as the central mediators of ECM remodeling throughout the metastatic cascade, particularly Membrane Type-1 MMP (MT1-MMP), MMP-2, and −9 [5–7]. Collectively, these MMPs play a key role in the degradation of the major structural components of the basement membrane (BM), which separates the primary tumour from the endothelium and is the first main structural barrier to metastasis [8, 9]. It was long thought that the role of MMPs was to mediate movement of cancer cells through the BM via their ability to degrade ECM components; however, MMPs are now known to be more pleiotropic than previously thought [10], participating in a multitude of cell processes during cancer progression [11, 12], and perhaps also acting as transcription factors [13–15],

MT1-MMP (MMP-14) is multifunctional MMP that affects cell behavior via proteolytic and non-proteolytic mechanisms [16, 17]. As a result of its proteolytic activity, MT1-MMP can cleave ECM and non-ECM substrates [5, 18], and participates in activation of the pro-forms of MMP-2 and MMP-9 [19, 20]. MT1-MMP also signals through the ERK and AKT pathways [21, 22] and mediates HIF-1α stabilization [23] via non-proteolytic mechanisms. Moreover, it has been shown that MT1-MMP overexpression also increases the migratory ability of breast cancer cells independent of its proteolytic activity [24, 25]. The mechanism underlying this increase in migratory potential remains poorly understood, with evidence suggesting that it involves ERK activation and cleavage of the cell adhesion molecule CD44 [26, 27]. However, reported data reveal contradictions regarding the requirement for TIMP-2 in increasing migratory potential, as some report the necessity for TIMP-2 to increase migration of cancer cells overexpressing MT1-MMP [24, 25], whereas others did not using similar cell models [26–28]. Other experimental inconsistencies regarding the source of MMPs, whether they are predominately stromal or cancer cell derived [29], and their role in mediating cancer cell growth and invasion in vivo are also under intense debate [7, 9, 30, 31].

To address this, we overexpressed MT1-MMP in MCF-7 breast cancer cells, which represent early stage breast cancer [32] and are naturally MT1-MMP deficient [33, 34], and for these reasons are commonly used by others to understand the effects of MT1-MMP overexpression [21, 24, 25, 27, 28]. We determined that high levels of MT1-MMP expression did not correlate with migratory potential, and rather that low levels of MT1-MMP are optimal to enhance migration. Similarly, we showed that overexpression of MT1-MMP in MDA-MB-231 cells, which are naturally invasive and express endogenous MT1-MMP [32, 34, 35], decreased their migratory potential and viability rather than enhanced it. These observations are consistent across platforms, both ex vivo with 3D cell culture [36], and with in vivo tumourigenesis and cancer cell extravasation assays [37], which all demonstrate that low MT1-MMP expression is optimal to induce a protrusive phenotype, increased invasiveness and metastatic capability in vivo*.* Additionally, we analyzed the level of MT1-MMP protein in human 21 T breast cancer cell lines, which represent a progression from atypical ductal hyperplasia (ADH) to invasive mammary carcinoma (IMC), to show that the metastatic cell line produces little MT1-MMP protein, consistent with our conclusions using MCF-7 and MDA-MB 231 breast cancer cells. This “low MT1-MMP” migratory phenotype is accompanied by concomitant levels of TIMP-2, thus reconciling many conflicting studies on proteolytic factors in primary human tumours.

## Methods

### Cell culture

MCF-7, MDA-MB 231 and HS578t human breast cancer cell lines were obtained from the American Type Culture Collection (Manassas, VA). Cells were maintained in DMEM/F-12 media (Thermo Fisher) supplemented with 10 % FBS, 100 IU/ml penicillin, 100 μg/ml streptomycin, and incubated at 37 °C and 5 % CO_2_.

### cDNA clones and reagents

Human MT1-MMP (sc116990), TIMP-2 (sc118083) and MMP-2 (sc321560) cDNA clones were purchased from Origene and subcloned into the vector pcDNA 3.3 (Thermo Fisher). The generation of the ALA + TIMP-2 cDNA construct in pcDNA 3.3 is described in Walsh et al. [38]. The following reagents were used: Recombinant human TIMP-2 and 4-aminophenylmercuric acetate (APMA) (Sigma-Aldrich), BB-94 (Batimastat), U-0126, and AKT inhibitor IV (Santa Cruz), and Furin inhibitor II (Millipore).

### Antibodies

For immunoblot analysis, the following primary antibodies were used: MT1-MMP (1:1000, AB6004, Millipore); MT1-MMP (1:1000, AB51074, Abcam); Phospho-ERK1/2 (1:2000, D13.14.4E), ERK1/2 (1:2000, 137 F5) (Cell Signaling Technology); TIMP-2 (1:1000, 3A4), β-Actin (1:1000, C4), and phospho-histone-3 (PH3) (1:5000, C1513) (Santa Cruz). Goat anti-mouse IgG (H + L) (Bio-Rad) and goat anti-rabbit IgG (H + L) (Thermo Fisher) HRP conjugates were used as secondary antibodies (1:10000). For immunofluorescence analysis we used MT1-MMP antibody AB6004 (1:200), and anti-rabbit-IgG-Alexa488 or Alexa594 (Thermo Fisher) as secondary antibodies (1:400).

### Transfection and generation of stable cell lines

MCF-7 and MDA-MB 231 cells were seeded at a density of 5×10^5^ cells/ml and incubated for 24 h. Following incubation, cells were transfected with Lipofectamine 2000 (Thermo Fisher) according to the manufacturer’s instructions. For transient transfection experiments, cells were incubated for 24 h after transfection and then utilized for experiments.

Stable cell lines were generated by transfection of cells with the respective cDNAs in the vector pcDNA 3.3, which contains a neomycin mammalian selection marker. Following transfection, cells were split 1:1000 and incubated in media containing 1 mg/ml G-418 (VWR). Individual colonies were selected after four weeks of incubation in selection media and expanded to assay for the levels of MT1-MMP by qPCR and immunoblotting. Stable cells lines expressing an shRNA sequence targeting MT1-MMP in the vector pRS (TR311445, Origene) were generated in the same manner expect using puromycin (2 μg/ml) as the selection antibiotic.

For zsGreen infection, cells were seeded at ~ 40 % density in a 6-well cell culture dish in 3 ml of media with a final concentration of 8 μg/ml polybrene and infected with 250 μL of virus. For virus production, the pLVX-ZsGreen1-N1 lentiviral plasmid was used. Twenty-four hours post-infection, the media containing virus was removed and replaced with puromycin selection media (2 μg/ml) for three days of incubation to select for infected cells.

### Generation of MMP-2, TIMP-2 and ALA + TIMP-2 conditioned media (CM)

Conditioned media (CM) containing high levels of MMP-2, TIMP-2, and ALA + TIMP-2 protein was created by transfecting MCF-7 cells with cDNA constructs coding for the respective proteins. Following a 24-h incubation post-transfection, transfected cells were washed with phosphate buffered saline (PBS) and incubated in DMEM/F12 media without FBS for 24 h. The serum-free CM was then collected, aliquoted and stored for later use. Conditioned media from mock-transfected cells was used as a control.

### Quantitative real-time PCR

RNA was collected from cells using the RNeasy Kit (Qiagen) and cDNA was synthesized from 1 μg of RNA using qScript cDNA supermix (Quanta). MT1-MMP mRNA levels were assayed by qPCR using PerfeCta SYBR Green Supermix (Quanta) and a CXF connect real time system with CFX manager software (Bio-Rad). mRNA levels were quantified by the ΔΔCT method and are displayed as fold change relative to parental MCF-7 cells. The level of GAPDH mRNA was used as the internal control. Primers are as follows: MT1-MMP; F: gcagaagttttacggcttgca, R: tcgaacattggccttgatctc, GAPDH; F: acccactcctccacctttga, R: ctgttgctgtagccaaattcgt [33].

### Immunoblotting

Post-incubation cells were washed 3× with PBS (pH = 7.2) and disrupted using lysis buffer (150 mM NaCl, 1 % NP-40, 0.5 % NaDC, 0.1 % SDS, 50 mM Tris pH 8.0) supplemented with protease/phosphate inhibitor (Thermo Scientific). Cell lysates were homogenized by sonication and 15 μg aliquots were analyzed by immunoblotting with MT1-MMP, TIMP-2, pH-3, β-actin, pERK1/2 or ERK1/2 primary antibodies, followed by incubation with the appropriate secondary HRP-conjugated antibody and detection using SuperSignal West Pico chemiluminescent substrate (Thermo Fisher). Protein lysate from human 21 T cell lines were acquired as previously described [39].

### Gelatin zymography and reverse zymography

Gelatin zymography and reverse zymography was done using samples of serum-free media (15 μl) as described previously [40]. To assess the proMMP-2 activation ability of MCF-7 cells, which endogenously express very low levels of MMP-2 [34], proMMP-2 CM was added to cells at a dilution of 30 μl proMMP-2 CM/mL SF media. For reverse zymography, serum-free media conditioned for 24 h by HS578t cells was used as the source of active MMP-2 within the gel, as these cells naturally express high levels of MMP-2 [33].

### Immunofluorescence

Samples for fluorescent gelatin degradation and 3D culture experiments were fixed and prepared for immunofluorescence according to their respective protocols (see below). MT1-MMP primary antibody (AB6004) was detected using anti-rabbit IgG Alexa594 or Alexa488 for Oregon green-488 gelatin degradation or 3D culture experiments respectively. F-actin was stained with Alexa633 phalloidin (1:100, Thermo Fisher) and nuclei with 4′,6-diamidino-2-phenylindole (1 μg/ml, BioShop Canada). Samples were imaged using a Nikon A1R+ confocal microscope (1.2 au) with a 20× dry or 60× oil-immersion lens and presented using NIS Elements software.

### Transwell assays

The migratory potential of cells was measured using 24-well 8 μm pour transwell inserts (Corning Costar). Cells (2×10^4^) were seeded on the upper chamber of the transwell in serum-free media and allowed to migrate towards the bottom chamber which was placed in DMEM/F-12 media supplemented with 10 % FBS. Migration assays were done with uncoated transwell inserts, whereas invasion assays were done with inserts coated with 20 % Matrigel. Cells that migrated to the lower chamber of the transwell insert were quantified as described previously [41]. Migrated cells were normalized to MCF-7/MDA-MB 231 parental cells in control conditions and are presented as a mean percentage ± SEM.

### Scratch closure migration assay

Cells were seeded at a density of 1×10^6^ cells in a 35 mm cell culture dish and allowed to form a monolayer for 24 h. Following incubation, media was removed and a scratch was made down the middle of the cell culture dish with a 100 μl pipette tip. Cells were washed 3× with PBS (pH 7.2) to remove cell debris and then incubated with fresh media. After 2 h, 10 images were captured down the length of the scratch that represent the ‘initial’ size of the scratch for that sample. Every 24 h for 3 days, the same area of the scratch was imaged to examine how cells migrated to close the scratch. Scratch closure was quantified using ImageJ 2.0.0 software by measuring the width of the scratch each day and normalizing it to the initial size of the scratch. Scratch closure is presented as a mean percentage of the initial scratch size ± SEM.

### Celltiter96® proliferation assay

Cells were seeded in triplicate (5000 cells/well) onto a 96-well cell culture dish in media supplemented with 10 % FBS or serum-free media. Immediately after seeding, 20 ul of Celltiter96® AQueous One Solution (Promega) was added in triplicate to each sample to obtain an initial reading and ensure that the different cell lines were seeded equally. The OD at 490 nm was measured using a Bio-Rad model 3550-UV microplate reader after incubating the cells with the substrate for 2 h. The substrate was added at the indicated day intervals and measured in the same way as the initial measurement to create a growth curve over that span, or to compare the effect of chemical inhibitors on cell viability during prolonged serum-free incubation.

### Fluorescent gelatin degradation assay

Coverslips were coated with either Oregon Green-488 gelatin (for IF analysis) or gelatin labeled using an Alexa594 protein labeling kit (for live imaging analysis) (Thermo Fisher) and used to analyze ECM degradation as per Martin et al. [42]. Immunofluorescence conditions are as described above.

### Three-dimensional (3D) cell culture

Cells (2.5×10^4^) were embedded in Matrigel (Corning Costar) and processed for immunofluorescence as per Cvetkovic et al. [43]. To quantify colony morphologies observed in 3D culture, five random 50 μm Z-stacks (2 μm step size) were acquired using DIC microscopy at 10× magnification every day for 5 days post-embedding. For each individual Z-stack, invasive features (disseminations and protrusions, see text) were blindly counted and normalized to the number of circular colonies per field of view. Immunofluorescence procedure was done as described above using 3 % BSA/PBS as the blocking buffer. Single cells (marked by DAPI), F-actin disseminations, F-actin protrusions and zsGreen protrusions were blindly counted from 20× 3D volumes and normalized to the number of circular colonies per field of view.

### Live-imaging and timelapse movies

Cells were embedded in Matrigel or seeded on Alexa594 gelatin coverslips as described above and placed in a live imaging chamber mounted on the stage of a Leica DM16000 B fluorescent microscope. To analyze the relationship between ECM degradation and migration, cells stably expressing zsGreen were incubated on Alexa594 gelatin coverslips and imaged at the same stage position at 10× magnification every 10 min for 20 h. These images were then compiled into timelapse movies using ImageJ. The zsGreen channel timelapse movies were used to quantify the migration of individual cells in an automated manner using the ADAPT plugin for ImageJ [44]. The ADAPT plugin (v1.146) was used with default conditions and the trajectory visualization output was used to group cells according to the distance migrated. The Alexa594 gelatin channel was used to manually quantify the percentage of cells that had degraded the underlying gelatin at different time points. To visualize the dynamics of cells in 3D culture, cells were embedded in Matrigel and z-stacks (100 μm, 5 μm step size) were acquired at 20× magnification every 30 min for 72 h. Focal panels showing colony features with the greatest clarity were isolated and compiled into timelapse movies using ImageJ.

### Avian embryo CAM implantation and extravasation efficiency assay

For implantation experiments, the superficial layer of the CAM of day 9 chicken embryos was removed to expose the underlying capillaries [45]. MDA-MB 231, MCF-7 or MCF-7 MT1-MMP cell lines stably expressing zsGreen were then resuspended in Matrigel and pipetted onto the exposed capillaries (5×10^5^ cells in 10 μl Matrigel/embryo). Eight days post-implantation the resulting tumour was imaged using a fluorescent stereoscope to examine vascularization of the tumour shown by the presence of non-fluorescent CAM vessels within the zsGreen tumour.

MCF-7 and MCF-7 MT1-MMP cell lines stably expressing zsGreen were used for intravenous injection into the CAM vasculature of day 14 chicken embryos and extravasation efficiency was quantified 24 h post-injection as per Kim et al. [37].

### Densitometry analysis

Quantitative analysis of immunoblots was done using QuantityOne software (Bio-Rad). Band intensity was obtained for the pERK and total ERK signal of each sample from three independent experiments. ERK activation is presented as a ratio between the pERK and the total ERK band intensity within each sample normalized to MCF-7 cells under control conditions.

### Statistics

Statistical analysis and graphing was performed using GraphPad Prism version 6.0 (GraphPad software, La Jolla, CA, USA). Data is presented as mean ± SEM. One-way ANOVA followed by Tukey’s post-hoc test was used unless otherwise indicated in the figure legend. Significant differences denoted by asterisks are shown versus MCF-7 or MDA-MB 231 parental cells in control conditions unless otherwise shown in figure. Different levels of statistical significance are denoted by a different number of asterisks and are as follows: ****p ≤ 0.0001, ***p ≤ 0.001, **p ≤ 0.01, *p ≤ 0.05. All experiments were repeated in triplicate with comparable results.

## Results

### Production of functional MT1-MMP in MCF-7 cells did not result in increased migratory potential

**Fig. 1:**
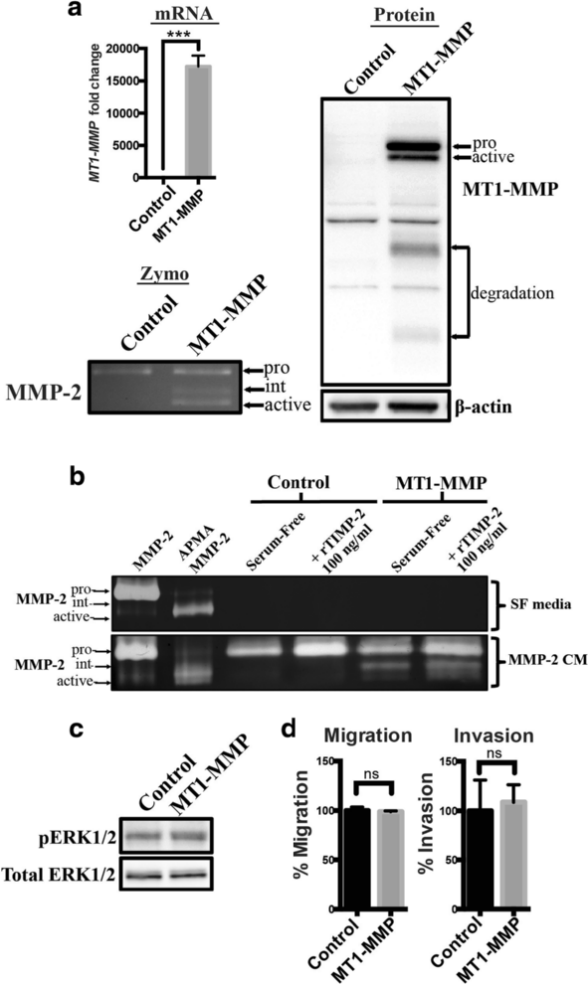
Transient overexpression of MT1-MMP in MCF-7 cells did not result in increased migration and invasion (**a**) qPCR, immunoblot, and gelatin zymography analysis of MT1-MMP mRNA, protein levels, and proMMP-2 activation ability, respectively, of MCF-7 breast cancer cells transiently transfected with MT1-MMP compared to mock transfected cells (control). Immunoblot analysis (AB6004) showed pro-, active, and degradation forms of MT1-MMP protein in MT1-MMP transfected MCF-7 cells. β-actin was used as a loading control. Gelatin zymography analysis showed that MCF-7 cells transiently transfected with MT1-MMP were capable of activating proMMP-2 after 24 h of incubation as shown by intermediate and active forms of MMP-2. **b** Gelatin zymography analysis of MCF-7 cells transiently transfected with MT1-MMP and incubated for 12 h with serum-free media (SF, top gel) or MMP-2 conditioned media (CM, bottom gel). Lanes 1 and 2: Controls showing proMMP-2 CM chemically activated by APMA. Lanes 4 and 6: Recombinant TIMP-2 (rTIMP-2) was added at 100 ng/ml to enhance MT1-MMP-mediated proMMP-2 activation. **c** Immunoblot analysis of MT1-MMP transfected cells showing phospho-ERK1/2 levels. Total ERK1/2 was used as a loading control. **d** Transwell migration and invasion assays of MCF-7 cells transiently transfected with MT1-MMP. Number of migrated/invaded cells were normalized to control MCF-7 cells and expressed as a mean percentage ± SEM. (ns, *p* > 0.05 by student’s *t*-test)

We transiently transfected MCF-7 breast cancer cells with wild-type untagged MT1-MMP cDNA and assessed proMMP-2 activation, ERK activation, migration and invasion (Fig. 1). qPCR and immunoblot analysis of MT1-MMP mRNA and protein levels, respectively, of MCF-7 cells transiently transfected with MT1-MMP cDNA showed that 24-h post-transfection these cells have very high levels of MT1-MMP mRNA (~17,000 fold relative to mock transfected cells) and produced predominately the pro- but also active isoform, along with degradation forms, of MT1-MMP protein (Fig. 1a). Gelatin zymography analysis demonstrated that MCF-7 cells transiently transfected with MT1-MMP are capable of activating proMMP-2 after 24 h of incubation with MMP-2 CM, as shown by the transition to the intermediate and active isoforms of MMP-2. Parental MCF-7 cells, which do not secrete endogenous proMMP-2, did not increase MMP-2 production after transfection with MT1-MMP, as determined by gelatin zymography (Fig. 1b, top gel). Addition of proMMP-2 conditioned media to transfected cells demonstrated that MT1-MMP overexpression activated proMMP-2 after 12 h of incubation, shown by the presence of an intermediate band, whose activation was enhanced by the addition of low levels of TIMP-2 (100 ng/ml) that resulted in the presence of more active MMP-2 isoform (Fig. 1b, bottom gel). Differing from published reports [24, 27, 28] we found no difference in ERK activation (Fig. 1c) or migration and invasion (Fig. 1d, ns > 0.05) in MCF-7 cells that produced functional MT1-MMP protein after transient transfection.

**Fig. 2:**
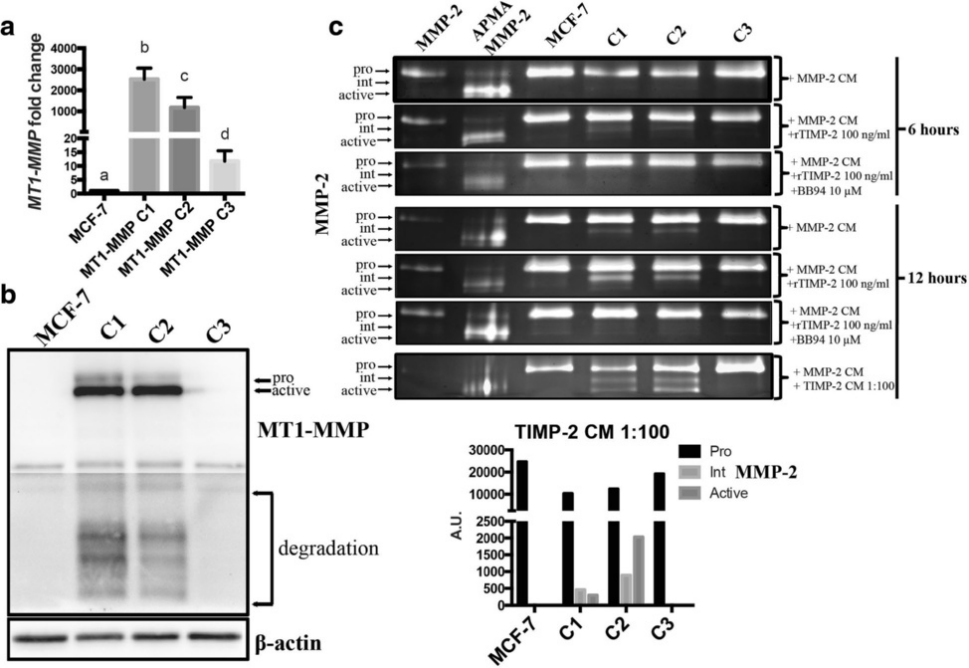
MCF-7 cell lines producing high levels of MT1-MMP protein demonstrated TIMP-2-mediated proMMP-2 activation (**a**) qPCR analysis of MT1-MMP mRNA from MCF-7 MT1-MMP cells lines that stably express different levels of MT1-MMP. Different letters indicate significant differences at *p* ≤ 0.05 by one-way ANOVA, Tukey’s post-hoc test. **b** Immunoblot analysis (AB51074) showing pro-, active, and degradation forms of MT1-MMP protein in MCF-7 MT1-MMP cell lines. β-actin was used as a loading control. **c** Gelatin zymography analysis of MCF-7 MT1-MMP cell lines incubated for 6 or 12 h with either MMP-2 CM alone, or in combination with rTIMP-2 at 100 ng/ml, or rTIMP-2 and BB94 (10 μm). Cells were also incubated for 12 h in MMP-2 CM supplemented with TIMP-2 CM diluted 1:100 in SF media (*bottom*). Bar graph shows densitometry quantification of MMP-2 isoforms from representative zymography of MCF-7 MT1-MMP cell lines incubated with MMP-2 CM and TIMP-2 CM diluted 1:100

To test whether the very high and heterogeneous levels of MT1-MMP expression achieved using transient transfection was a factor behind the discrepancies between our observation and other reports, we created clonal MCF-7 cell lines that stably express significantly different levels of MT1-MMP (Fig. 2). These cell lines, MCF-7 MT1-MMP C1, C2, and C3 cells (hereby referred to as C1, C2 or C3 cells), significantly differ in their stable expression of MT1-MMP transcript from high (C1, ~2,500 fold compared to MCF-7 parental cells), medium (C2, ~1100 fold) to low (C3, ~11 fold) as determined by qPCR analysis (Fig. 2a). Immunoblot analysis of these MCF-7 MT1-MMP cell lines showed that both pro- and active- isoforms of MT1-MMP protein were detected in C1 cells, whereas the active isoform was predominantly detected in C2 cells and no MT1-MMP protein could be detected by immunoblot in C3 cells (Fig. 2b). Cell lines where active MT1-MMP was detected via immunoblot (C1 and C2 cells) were capable of activating proMMP-2 in a time-dependent manner, and as expected this was enhanced by low levels of recombinant TIMP-2 (100 ng/ml), shown by a transition to the intermediate form of MMP-2, which was completely inhibited by addition of BB94 (10 μM) (Fig. 2c). Addition of diluted (1:100) TIMP-2 CM demonstrated a greater enhancement of proMMP-2 activation by C1 and C2 cells as shown by the presence of both intermediate and active forms of MMP-2. Densitometry quantification of a representative zymography with diluted TIMP-2 CM demonstrates that C2 cells have the highest ability to activate proMMP-2, as shown by increased levels of intermediate and active forms, despite not expressing the highest level of MT1-MMP (Fig. 2c, bottom graph). Active MMP-2 was not detected in C3 cells.

**Fig. 3:**
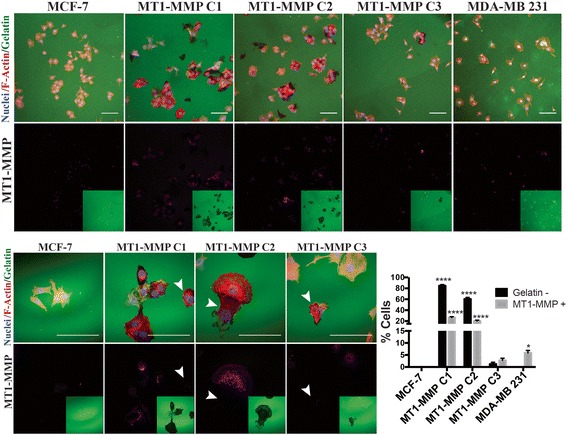
MCF-7 cell lines that express high levels of MT1-MMP demonstrated widespread ECM degradation (a) MCF-7, MT1-MMP cell lines, and MDA-MB 231 breast cancer cells were incubated on Alexa488 gelatin-coated coverslips for 24 h and processed for immunofluorescence to examine cytoplasmic MT1-MMP protein and ECM degradation. Representative fields of view are shown at 20× (*top panels*) and 60× (*bottom panels*) magnification. Panels are composed of an overlay showing the nuclei, F-actin, and Alexa488 gelatin signal (*top*) and the MT1-MMP signal with an inset of the Alexa488 gelatin channel (*bottom*). White arrows indicate cells that have degraded the underlying gelatin but are devoid of MT1-MMP signal. Scale bars = 100 μm. Cells in each sample positive for cytoplasmic MT1-MMP protein signal (MT1-MMP +) or devoid of underneath Alexa488 gelatin signal (Gelatin -) were quantified per 20× fields of view and are shown as mean percentage of total cells per field of view ± SEM

To confirm that C3 cells produced MT1-MMP protein (not detectable by immunoblot) we used an imaging approach whereby cells were seeded on Oregon-green 488 gelatin coated coverslips and examined using immunofluorescence for both MT1-MMP protein presence and degradation of the underlying fluorescently labeled ECM [42]. MDA-MB 231 breast cancer cells were also used in this analysis to compare MCF-7, C1, C2 and C3 cells to a cell line demonstrated to express naturally increased levels of MT1-MMP (Fig. 3). Quantification of fields of view acquired at 20× magnification demonstrated no detectable cytoplasmic MT1-MMP immunological signal nor gelatin degradation in parental MCF-7 cells. In contrast, 84 and 60 % of C1 and C2 cells displayed ECM degradation (gelatin -), respectively, with approximately 20 % of these cells also displaying punctate cytoplasmic immunological signal for MT1-MMP protein (Fig. 3, bar graph). A low percentage of MT1-MMP C3 cells (~1.5 %) showed ECM degradation, and a higher percentage (~3 %) showed a cytoplasmic MT1-MMP signal, although these amounts were non-significantly different than parental MCF-7 cells. Interestingly, in all conditions where MT1-MMP was overexpressed, there were cells that had degraded the underlying ECM that, paradoxically, were devoid of MT1-MMP protein signal (Fig. 3, white arrows). MDA-MB 231 breast cancer cells were not capable of widespread cell-associated ECM degradation, and only ~6 % of cells displayed cytoplasmic MT1-MMP signal. Taken together with the data in Fig. 2, these analyses show that ECM degradation and proMMP-2 activation is associated with elevated MT1-MMP expression levels and immunodetection of MT1-MMP protein (C1 and C2), consistent with other reports. However, there is also an inconsistency when comparing these results to C3 and MDA-MB 231 cells, as C3 cells display no detectable levels of MT1-MMP protein via immunoblot, but some C3 cells did degrade the underlying ECM and displayed cytoplasmic MT1-MMP protein via immunofluorescence. Additionally, the pattern of ECM degradation and cytoplasmic MT1-MMP protein in MDA-MB 231 cells is most consistent with those of C3 cells, suggesting that cells that express low levels of MT1-MMP acquire invasive capabilities, despite reports that suggest high levels of MT1-MMP are typically needed for such phenotypes [24, 27, 28].

### Low levels of MT1-MMP expression combined with high levels of TIMP-2 increased the migratory potential of MCF-7 cells via the ERK pathway

As our analysis using transient transfection of MT1-MMP demonstrated no change in cell migration, we wanted to further examine this migratory effect as well as the potential role of ERK activation using our stable cell lines that express lower levels of MT1-MMP than transiently transfected cells. As others have previously shown that TIMP-2 can modulate rapid ERK activation by MT1-MMP expressing breast cancer cells [24, 25], we utilized conditioned media (CM) that contained high levels of TIMP-2, or ALA + TIMP-2 (a TIMP-2 mutant that cannot inhibit MMPs enzymatic activity, but which has been shown to still bind cell surface MT1-MMP and upregulate ERK activation [24, 46]) to test this parameter. Analysis of these CMs via immunoblot showed that high but equal levels of TIMP-2 and ALA + TIMP-2 are present in the media, whereas reverse zymography analysis demonstrated that only TIMP-2 was capable of inhibiting MMPs (Additional file 1: Figure S1a). Using these CMs, we tested the ability of MCF-7 MT1-MMP cell lines to sequester TIMP-2 from the media by treating these cells with diluted CM. TIMP-2 levels remained equal after 15 min, but after 12 h of incubation TIMP-2 levels in the media decreased corresponding to the level of MT1-MMP expression (Additional file 1: Figure S1b), further confirming that these cells produced differing levels of functional cell surface MT1-MMP. Quantification of TIMP-2 protein in TIMP-2 CM via immunoblot was performed which demonstrated that undiluted TIMP-2 CM contains approximately 10 μg/ml TIMP-2 protein (Additional file 1: Figure S1c). The proMMP-2 activation ability of MCF-7 MT1-MMP cells was also tested in the presence of increasing levels of TIMP-2 or ALA + TIMP-2 CM by gelatin zymography and reverse zymography (Additional file 1: Figure S1d). Only C1 and C2 cells were capable of activating proMMP-2, and this was enhanced by addition of low levels of TIMP-2 (~100 ng/ml), but inhibited by high levels of TIMP-2 (> ~1 μg/ml) [25, 47, 48], consistent with the observations of others that TIMP-2 at 100 ng/ml is optimal for proMMP-2 activation by MT1-MMP expressing cells [49]. ALA + TIMP-2 did not enhance proMMP-2 activation in these cells consistent with the requirement for both the N- and C-terminal domains of TIMP-2 to act as an adapter for proMMP-2 [20]. Taken together, this analysis confirmed the functionality of our TIMP-2/ALA + TIMP-2 CMs and demonstrated how MCF-7 cells producing high levels of MT1-MMP protein follow the well-defined mechanism described for MT1-MMP/TIMP2 mediated activation of proMMP-2.

**Fig. 4:**
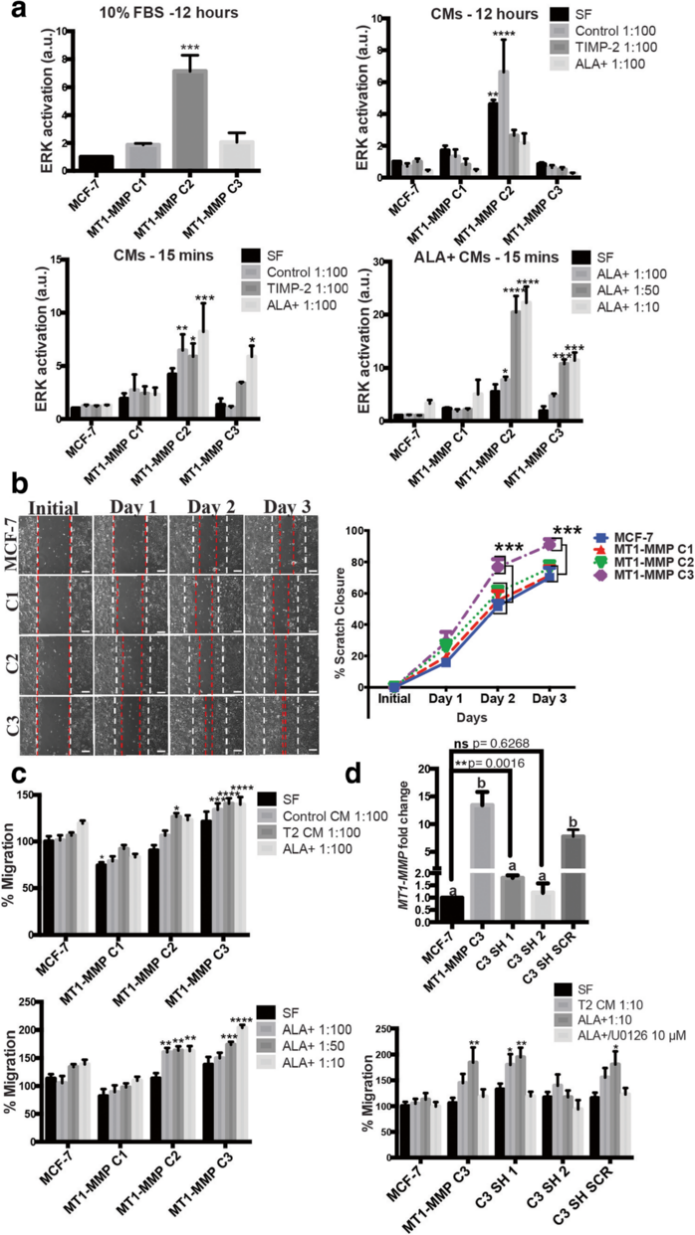
Low MT1-MMP/high TIMP-2 was optimal to promote migration and ERK activation in MCF-7 cells. **a** ERK activation in MCF-7 and MT1-MMP cells after incubation for 12 h (top) or 15 min (*bottom*) in media containing 10 % FBS or different dilutions of TIMP-2 or ALA + TIMP-2 CM in SF media. **b** Scratch closure migration assay of MCF-7 MT1-MMP cell lines monitored for 3 days. Shown are representative 10× fields of view. The white dotted lines indicate the initial scratch size; red dotted lines indicate the scratch size at the respective day. Scale bars = 100 μm. Line graph on the right shows scratch closure quantification that demonstrates significantly increased migratory potential of C3 cells. **c** Transwell migration assays of MCF-7 MT1-MMP cell lines incubated for 48 h in TIMP-2, or ALA + TIMP-2 CM diluted 1:100 (*top*), or ALA + TIMP-2 CM in increasing dilutions (*bottom*). **d** (*top*) qPCR analysis showing MT1-MMP mRNA from two cell lines derived from MT1-MMP C3 cells that stably express an shRNA construct targeting MT1-MMP, and one cell line stably expressing a control scrambled shRNA construct. Different letters indicate significant differences at *p* ≤ 0.05 by one-way ANOVA, Tukey’s post-hoc test. Individual student’s t-tests comparing MCF-7 cells against the C3 SH 1 cell line is also shown. (*bottom*) Transwell migration assay of MT1-MMP C3 cell lines incubated for 48 h in either TIMP-2 or ALA + TIMP-2 CM diluted 1:10, or ALA + TIMP2/U0126 (10 μm)

To then examine the level of ERK activation by MCF-7 MT1-MMP cell lines and the role of TIMP-2 in mediating this process, cells were incubated in media containing either 10 % FBS or in serum-free (SF) media containing different dilutions of TIMP-2 or ALA + TIMP-2 CM. Phospho-ERK levels were then examined using immunoblotting (Additional file 2: Figure S2) and quantified via densitometry (Fig. 4a). In media containing 10 % FBS, only C2 cells had a significantly higher level of phospho-ERK compared to parental MCF-7 cells. A 12-h incubation with TIMP-2 or ALA + TIMP-2 CM demonstrated a significant decrease in ERK activation in C2 cells. In contrast, a short 15 min incubation period with CMs demonstrated a significant increase in ERK activation of C2 and C3 cells, but not C1 cells, particularly with ALA + TIMP-2 treatment. Additionally, short incubation periods with dilutions of ALA + TIMP-2 CM showed that ERK activation in C2 and C3 cells, but not C1 cells, is significantly upregulated by high levels of ALA + TIMP-2 protein. Overall, analysis of phospho-ERK levels demonstrated that cells expressing medium (C2) and low (C3) levels of MT1-MMP demonstrate increased ERK activation that is upregulated by high levels of TIMP-2 (> ~ 200 ng/ml) and independent of MMP inhibition.

We then examined how TIMP-2-mediated ERK activation related to the migratory potential of MCF-7 MT1-MMP cell lines. A scratch closure assay over a span of 3 days demonstrated that only C3 cells closed the scratch significantly faster than parental MCF-7 cells, indicating increased migratory ability of C3 cells (Fig. 4b). Migration was then examined using transwell assays where the cells were incubated in SF media containing TIMP-2 and ALA + TIMP-2 CMs in the upper compartment and allowed to migrate towards the lower compartment containing 10 % FBS. TIMP-2 CM caused a significant increase in the number of migrated C2 and C3 cells, while ALA + TIMP-2 CM also caused a significant increase in the number migrated C3 cells (Fig. 4c, top). Higher dilutions of ALA + TIMP-2 CM showed a similar trend whereby only C2 and C3 cells demonstrated a significant increase in the number of migrated cells (Fig. 4c, bottom). C1 cells, which express the highest level of MT1-MMP, demonstrated an inability to increase migration potential when incubated with TIMP-2 CMs, and this was consistent with the lack of ERK activation upon TIMP-2 treatment in these cells.

To determine if the enhanced migratory ability of C3 cells was a result of the relatively low (11 fold) increase in MT1-MMP expression, we transfected these cells with a construct expressing an shRNA targeting MT1-MMP, and selected two cells lines: C3 SH 1, a partial knockdown which displayed a small but significant increase in MT1-MMP mRNA level (1.8 fold change *p* ≤ 0.01) as compared to parental MCF-7 cells, and C3 SH 2, a complete knockdown with 1.1 fold change (ns *p* = 0.62). A cell line was also derived from C3 cells stably expressing a scrambled control shRNA (C3 SH SCR) (Fig. 4d, top). Migration of these C3 cell line variants were assayed during incubation in SF media containing TIMP-2 or ALA + TIMP-2 CM, or ALA + TIMP-2 CM along with the ERK inhibitor U0126 (10 μM). Consistent with the previous transwell assays, C3 and control C3 SH SCR cells were significantly more migratory than parental MCF-7 cells when exposed to high levels of TIMP-2 (> ~1 μg/ml), and this correlated with ERK activation as ALA + TIMP-2/U0126 incubation attenuated this migration (Fig. 4d, bottom). This analysis also showed that complete knockdown of MT1-MMP expression (C3 SH 2) inhibited the TIMP-2-mediated increase in migration, indicating that MT1-MMP is needed for enhancement of migratory potential via TIMP-2. Surprisingly, C3 SH 1 cells, which express a small but significant (1.8 fold) increase in MT1-MMP expression, demonstrated a similar TIMP-2-mediated increase in migration via ERK activation. Additionally, we attempted to knockdown MT1-MMP expression in C1 and C2 cells to rescue migratory potential using the same approach, however, this was unsuccessful using transfection of shRNA (not shown) presumably because of the excessively high expression of MT1-MMP in these cells. Taken together, this data demonstrated that TIMP-2 enhancement of migratory potential is indeed dependent on MT1-MMP and ERK activation, but low levels (1.8 -11 fold) of MT1-MMP expression appear to be optimal to increase migratory ability, whereas high (> ~1000 fold) MT1-MMP expression does not result in augmented cell migration.

### Low levels of MT1-MMP expression and activity were optimal for cell viability in serum free conditions

**Fig. 5:**
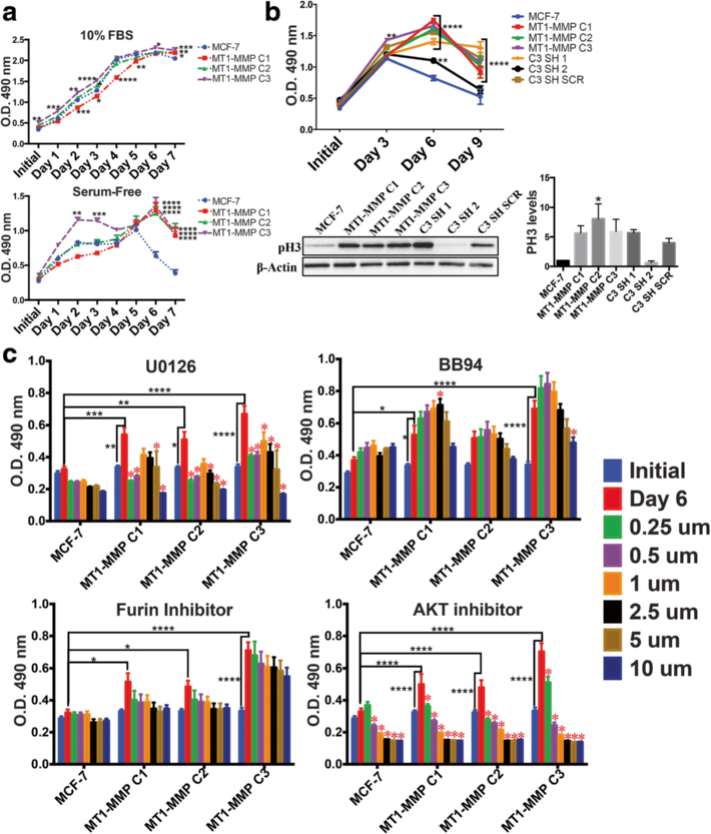
Low levels of MT1-MMP expression increased survivability of MCF-7 breast cancer cells to serum-free stress. **a** Viability of MCF-7 MT1-MMP cell lines during incubation in media containing 10 % FBS (*top*) or serum free media (*bottom*) measured daily for 7 days using Celltiter96®. **b** Viability of MCF-7 MT1-MMP cell lines and C3 cell line variants during incubation in SF media measured every 3 days for 9 days. Immunoblot analysis (*bottom*) shows PH3 protein levels collected from MCF-7 MT1-MMP cell lines and C3 cell line variants incubated in SF media for 6 days. β-actin was used as a loading control. Bar graph (*right*) shows densitometry analysis of PH3 levels. **c** Viability of MCF-7 MT1-MMP cell lines was measured after incubation for 6 days in SF media containing increasing concentrations of U0126, BB94, a furin inhibitor or an AKT inhibitor. Black asterisks show statistically significant differences between the initial and day 6 viability within cell lines, and also differences between the day 6 viability of MCF-7 cells compared to MT1-MMP expressing cell lines. Red asterisks indicate significant differences (*p* ≤ 0.05) within cell lines between the day 6 viability in SF media compared to the viability after 6 days of incubation with the different concentrations of the inhibitors

To analyze how MT1-MMP overexpression mediated survivability and how this might explain the different migratory potential of MCF-7 MT1-MMP cell lines, we measured cell viability using CellTiter 96® AQueous One Solution each day for 7 days during incubation in media containing 10 % FBS or SF media, which represents the two compartments in the transwell assay. In 10 % FBS, C1 cells (high MT1-MMP level) showed a significantly lower viability throughout the 7 days. Conversely C3 cells (low MT1-MMP level) were significantly more viable from seeding until day 3 (Fig. 5a, top). Similarly, in SF media C1 cells were less viable than parental MCF-7 cells from seeding until day 5, whereas C3 cells were significantly more viable during this time (Fig. 5a, bottom). After 6 days of incubation in SF media, all MT1-MMP expressing cells were significantly more viable than MCF-7 cells indicating that MT1-MMP has an essential role in mediating survivability during prolonged absence of growth factors. To confirm that MT1-MMP was responsible for this increased viability during SF incubation, we repeated the analysis using the C3 knockdown variants. Consistent with the previous analysis, only C3 cells had significantly higher viability at day 3, but by day 6 and 9 all MT1-MMP expressing cell lines were significantly more viable than MCF-7 cells (Fig. 5b). Viability measurements of the C3 knockdown variants demonstrated that MT1-MMP is responsible for enhanced viability during SF incubation as C3 SH 2 cells, which are MT1-MMP deficient similar to parental MCF-7 cells, are less viable than other cell lines at day 6, and show the same viability as MCF-7 cells at day 9. To corroborate these results, we also analyzed cell lysate from all MCF-7 cell lines via immunoblot (Fig. 5b, bottom) for the cell proliferative marker phospho-histone 3 (PH3), and quantified this using densitometry (Fig. 5b, bar graph). All MCF-7 cell lines that expressed significantly higher levels of MT1-MMP than MCF-7 cells showed higher levels of PH3 after incubation in SF media, particularly C2 cells, which accumulated high levels of active MT1-MMP protein, and showed significantly higher levels of PH3 than MCF-7 cells.

We then examined how inhibition of select signaling pathways affected the viability of MCF-7 MT1-MMP cell lines during prolonged SF incubation to gain insight into the mechanism behind MT1-MMP-mediated survivability. The chemical inhibitors U0126, BB94, furin inhibitor II, and AKT inhibitor IV were used to examine their effect on the viability of MCF-7 MT1-MMP cell lines after 6 days of SF incubation. ERK inhibition and AKT inhibition significantly decreased the viability of all MT1-MMP expressing cells consistent with the observations of others [21, 24]. Furin inhibitor (which would inhibit intracellular activation of MT1-MMP) showed a non-significant dose dependent decrease in the viability of cells expressing MT1-MMP, a trend not seen in MCF-7 parental cells, indicating that high levels of pro-MT1-MMP likely negatively mediated viability. Unlike all the other inhibitors that generally decreased viability, low concentrations of BB94 trended to increase the viability of all MT1-MMP expressing cells, especially C1 cells which displayed a significant increase in viability after 6 days of SF incubation with 2.5 μM BB94 (Fig. 5c). Taken together, this data demonstrated that MT1-MMP mediated survivability to serum-free stress, but that excessive MT1-MMP proteolytic activity may be counterproductive to increased viability. Furthermore, this analysis also demonstrated that cell viability has to be considered as a component to the pattern of migration seen in transwell assays, as C3 cells which were the most migratory are also the most viable, whereas in contrast C1 cells were the least viable and migratory. Therefore, it is difficult to delineate the relative contribution of changes in cell proliferation or changes in migratory capability to the difference in the number of cells quantified in the bottom compartment of a transwell at the endpoint of the assay.

### MT1-MMP expression did not correlate with migratory potential of breast cancer cells

**Fig. 6:**
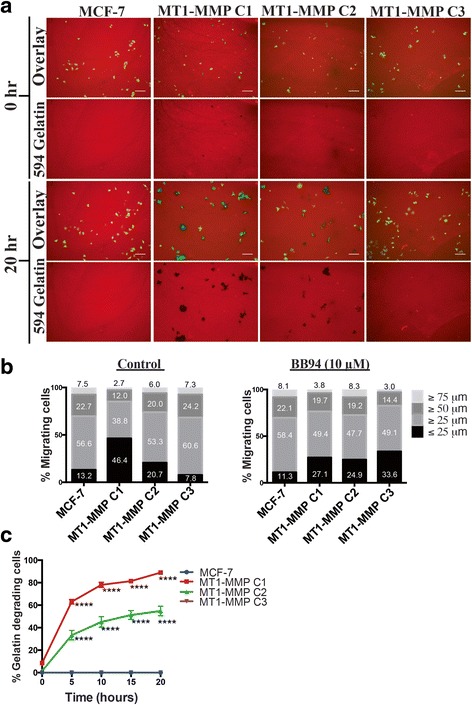
MT1-MMP activity is inversely correlated to the migratory potential of MCF-7 breast cancer cells (**a**) MCF-7 MT1-MMP cell lines stably expressing zsGreen were seeded on Alexa594-gelatin coated coverlips in media containing 0.1 % DMSO (control) or 10 μm BB94 and incubated in a live imaging chamber. Each sample was imaged at the same five stage positions every 10 mins for 20 h to visualize zsGreen cell movement and associated ECM degradation (Additional files 3, 4, 5 and 6). Shown are stills of the Alexa594 gelatin channel and an overlay including the zsGreen cells at time 0 and 20 h post-seeding of the control sample (BB94 not shown). Scale bars = 100 μm. **b** Time-lapse videos from (**a**) were analyzed using the ADAPT plugin for ImageJ and all individual cells tracked from each cell line were examined and grouped according to their migration distance from initial point of tracking. **c** Percentage of cells per field of view from each cell line that degraded the underlying AlexaFluour594-gelatin at five different time points


Here we present evidence that high MT1-MMP overexpression (transient transfectants, C1 and C2 cells) does not correlate with the migration of MCF-7 breast cancer cells, as seen using the transwell migration assay, which we showed also included a substantial cell viability component. Increased MT1-MMP-mediated viability may have yielded a higher number of cells in the transwell compartments, which may have resulted in incorrect conclusions about migratory potential if viability were not considered. So to confirm that high MT1-MMP overexpression (>1000 fold compared to MCF-7 cells) does not result in increased migration, we used a live imaging approach with automated migration quantification of MCF-7 MT1-MMP cells that were stably expressing fluorescent zsGreen protein and incubated on Alexa594 gelatin coated coverslips in control conditions (0.1 % DMSO) (Fig. 6a, Additional files 3, 4, 5 and 6) and with BB94 (10 μM) (data not shown). Automated quantification using the ADAPT plugin for ImageJ software [44] allowed us to track the migration of all individual cells in three independent experiments, which were then grouped according to their distance migrated (Fig. 6b, Additional file 7). This analysis showed that high MT1-MMP overexpression inhibited migration of MCF-7 cells as a higher proportion of C1 and C2 cells migrated a shorter distance from the initial point of tracking compared to parental MCF-7 cells, as 53 % and 79 % of C1 and C2 cells, respectively, migrated more than 25 μm, compared to 86 % of parental MCF-7 cells that migrated the same distance. In contrast, C3 cells did have increased migration ability compared to MCF-7 cells, as 92 % of C3 cells migrated more than 25 μm. Importantly, this 6 % increase in migratory ability between MCF-7 and C3 cells does not reflect the increase seen using the transwell assays (20-100 % increase), confirming that there is a cell viability component underlying the number of cells that transgressed to the bottom transwell compartment. BB94 incubation did not affect the migration of MCF-7 cells or C2 cells, but did alter the migratory patterns of C1 and C3 cells. Inhibition of MMP activity by BB94 decreased the migratory ability of C3 cells as 92 % of cells migrated more than 25 μm in control conditions compared to 66 % during BB94 incubation. C1 cells, which express the highest level of MT1-MMP and are the least migratory under control conditions, markedly improved their migratory potential during BB94 incubation as 73 % of cells migrated more than 25 μm (compared to 53 % in control conditions). This migration data is consistent with the cell viability assay during BB94 incubation, as C1 cells saw an augmentation rather than decrease in viability and migration when MMPs were inhibited, suggesting that excessive MMP activity is counterproductive to those cellular processes. Analysis of the percentage of cells that degraded the underlying ECM demonstrated a time-dependent increase in ECM degradation correlated to high MT1-MMP expression in C1 and C2 cells (Fig. 6c), confirming functional MT1-MMP protein production in these cells during the course of this assay. Incubation with BB94 (10 μM) did not allow for degradation of the fluorescent gelatin by MT1-MMP expressing cells (not shown). Taken together, this analysis demonstrated that low levels of MT1-MMP expression (11 fold compared to parental cells) are optimal for increased migratory ability of MCF-7 breast cancer cells, whereas high MT1-MMP overexpression (>1000 fold) does not increase migration but allows for widespread ECM degradation.

Additional files 3. Time-lapse analysis of ECM degradation and cell migration of MCF-7 MT1-MMP cells. Parental MCF-7 (Additional file 3), and MT1-MMP C1, C2, and C3 (Additional files 4, 5 and 6, respectively) cells stably expressing zsGreen (green) were seeded on Alexa594 gelatin coated coverslips (red) and incubated in a live imaging chamber at 37 °C, 5 % CO_2_. Images were acquired using a Leica DM16000 B fluorescent microscope. Frames were taken every 10 min for 20 h and compiled into time-lapse movies using ImageJ. Representative stills of each cell line at time 0 and 20 h are shown in Fig. 6. Scale bars = 100 μm. (AVI 11745 kb)



Additional file 4. Time-lapse analysis of ECM degradation and cell migration of MCF-7 MT1-MMP cells. Parental MCF-7 (Additional file 3), and MT1-MMP C1, C2, and C3 (Additional files 4, 5 and 6, respectively) cells stably expressing zsGreen (green) were seeded on Alexa594 gelatin coated coverslips (red) and incubated in a live imaging chamber at 37 °C, 5 % CO_2_. Images were acquired using a Leica DM16000 B fluorescent microscope. Frames were taken every 10 min for 20 h and compiled into time-lapse movies using ImageJ. Representative stills of each cell line at time 0 and 20 h are shown in Fig. 6. Scale bars = 100 μm. (AVI 17197 kb)



Additional file 5. Time-lapse analysis of ECM degradation and cell migration of MCF-7 MT1-MMP cells. Parental MCF-7 (Additional file 3), and MT1-MMP C1, C2, and C3 (Additional files 4, 5 and 6, respectively) cells stably expressing zsGreen (green) were seeded on Alexa594 gelatin coated coverslips (red) and incubated in a live imaging chamber at 37 °C, 5 % CO_2_. Images were acquired using a Leica DM16000 B fluorescent microscope. Frames were taken every 10 min for 20 h and compiled into time-lapse movies using ImageJ. Representative stills of each cell line at time 0 and 20 h are shown in Fig. 6. Scale bars = 100 μm. (AVI 14891 kb)



Additional file 6. Time-lapse analysis of ECM degradation and cell migration of MCF-7 MT1-MMP cells. Parental MCF-7 (Additional file 3), and MT1-MMP C1, C2, and C3 (Additional files 4, 5 and 6, respectively) cells stably expressing zsGreen (green) were seeded on Alexa594 gelatin coated coverslips (red) and incubated in a live imaging chamber at 37 °C, 5 % CO_2_. Images were acquired using a Leica DM16000 B fluorescent microscope. Frames were taken every 10 min for 20 h and compiled into time-lapse movies using ImageJ. Representative stills of each cell line at time 0 and 20 h are shown in Fig. 6. Scale bars = 100 μm. (AVI 18100 kb)



Additional file 7. ADAPT workflow for automated analysis of cell migration. Shown is an example of the ADAPT plugin and associated trajectory visualization of the MCF-7 MT1-MMP C2 video (Additional file 5). Additional file 5 is shown as the overlay of zsGreen cells (green) and Alexa594 gelatin coating (red), as well as the individual channels (top). The zsGreen channel was used for the ADAPT analysis (bottom, left) to yield a trajectory visualization of individual cells from initial point of tracking (bottom, right). Three videos from independent experiments were analyzed in this manner and the trajectory visualization from each was used to quantify individual cell migration after 20 h. Cell migration data is compiled in Fig. 6b. Scale bars = 100 μm. (MOV 5300 kb)


**Fig. 7:**
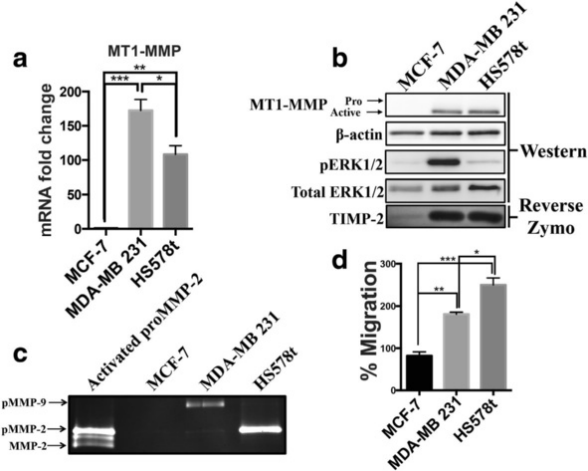
MT1-MMP expression does not correlate with increased migratory potential of breast cancer cells. **a** qPCR analysis of *MT1-MMP* mRNA levels from MCF-7, MDA-MB 231, and HS578t breast cancer cells. **b** Immunoblot (AB51074) and reverse zymography analysis comparing MT1-MMP, phospho-ERK and TIMP-2 protein levels between MCF-7, MDA-MB 231, and HS578t breast cancer cells. β-actin and total ERK1/2 were used as loading controls. **c** Gelatin zymography analysis of MCF-7, MDA-MB 231, and HS578t breast cancer cells incubated in SF media for 12 h. Lane 1 shows proMMP-2 CM activated by MCF-7 C2 cells treated with TIMP-2 CM diluted 1:100 to show proMMP-2 activation as a result of TIMP-2/MT1-MMP. **d** Transwell migration assay of MCF-7, MDA-MB 231, and HS578t breast cancer cells incubated in SF media for 24 h

To further investigate the correlation between MT1-MMP expression levels and the migratory potential of cells, we extended our observations to MDA-MB 231 and HS578T breast cancer cells by first assessing endogenous MT1-MMP levels in these cell lines. Both MDA-MB 231 and HS578t cells expressed significantly higher levels of MT1-MMP mRNA than MCF-7 cells (~172 and ~100 fold, respectively), and are significantly different from each other (Fig. 7a). Consistent with their high levels of expression, MDA-MB 231 and HS578t cells produce active MT1-MMP protein, and also have higher levels of phospho-ERK than MCF-7 cells. Reverse zymography analysis showed that MDA-MB 231 and HS578t cells also produce higher levels of TIMP-2 protein compared to MCF-7 cells (Fig. 7b). Gelatin zymography showed that MDA-MB 231 cells predominately secreted proMMP-9, whereas HS578t cells secreted high levels of proMMP-2 (Fig. 7c). Transwell migration assays demonstrated that MDA-MB 231 and HS578t were significantly more migratory than MCF-7 cells (Fig. 7d). Importantly, HS578t cells were significantly more migratory than MDA-MB 231 cells even though they express lower levels of MT1-MMP.

**Fig. 8:**
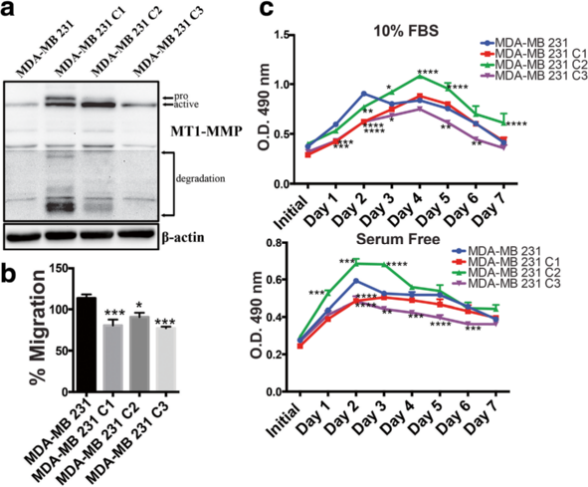
Overexpression of MT1-MMP in MDA-MB 231 cells negatively affected migration and viability (**a**) Immunoblot analysis (AB6004) showing pro-, active, and degradation forms of MT1-MMP in MDA-MB 231 breast cancer cells and three MDA-MB 231 cell lines expressing different levels of MT1-MMP*.* β-actin was used as a loading control. **b** Transwell migration assay of MDA-MB 231 MT1-MMP cells incubated in SF media for 12 h. **c** Viability of MDA-MB 231 MT1-MMP cell lines during incubation in media containing 10 % FBS (*top*) or serum free media (*bottom*) measured daily for 7 days using Celltiter96®

We then tested how overexpression of MT1-MMP in MDA-MB 231 cells affects the migration of cells that natively express MT1-MMP and have endogenous migratory ability. MDA-MB 231 MT1-MMP stable cell lines were created and three were selected which produce the isoforms of MT1-MMP shown via immunoblot (Fig. 8a). MDA-MB 231 C1 cells produce pro- and active- MT1-MMP, whereas MDA-MB 231 C2 and C3 produce predominantly active MT1-MMP at higher levels than parental MDA-MB 231 cells. Transwell migration analysis of these MDA-MB 231 MT1-MMP cell lines showed that overexpression of MT1-MMP significantly decreased their migratory potential compared to parental MDA-MB 231 cells (Fig. 8b). Viability of these cell lines was analyzed in 10 % FBS (top) and SF media (bottom) in the same manner as MCF-7 cell lines to show that MT1-MMP overexpression significantly inhibited viability, particularly of MDA-MB 231 C1 and C3 cells (Fig. 8c). Further, MDA-MB 231 C2 cells, which accumulate high levels of active MT1-MMP protein, exhibited increased viability in SF media and during prolonged incubation in 10 % FBS, which was consistent with both our PH3 analysis of MCF-7 C2 cells, and with their higher migration levels relative to the other MDA-MB 231 MT1-MMP cell lines. Taken together these analyses show that naturally increased MT1-MMP expression in transformed cancer cells (MDAMB-231, HS578t cells) does result in enhanced migration, but this is not the case for experimental MT1-MMP overexpression (MT1-MMP cell lines), as increasing overexpression of MT1-MMP decreases rather than enhances the migratory potential of breast cancer cells.

### Low level of MT1-MMP expression mediated a protrusive phenotype in 3D culture

**Fig. 9:**
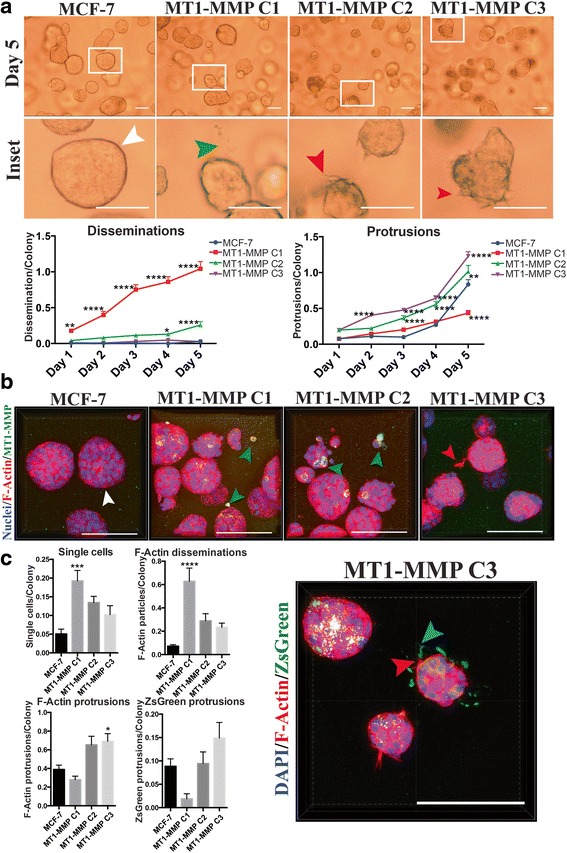
MT1-MMP overexpression in MCF-7 cells induced loss of colony organization and was inversely correlated with a protrusive morphology in 3D culture. **a** (*top*) MCF-7 MT1-MMP cells were embedded in Matrigel and imaged every day for 5 days at 10× magnification. Shown is a representative field of view of each cell line at day 5, and indicated inset images which show the cell features quantified: Circular colonies (*white arrow*), disseminations around colonies (*green arrow*), or protrusions emanating from colonies (*red arrows*). Scale bars = 100 μm. (*bottom*) Five z-stacks per cell line were acquired every day for 5 days and disseminations and protrusions were quantified per colony for each cell line. **b** Representative 3D volume views of immunofluorescence analysis after MCF-7 MT1-MMP cells were embedded in Matrigel for 5 days. Samples were imaged using confocal microscopy at 60× and are displayed as overlays showing MT1-MMP signal (*green*), DAPI (*blue*) and Alexa633 phalloidin (*red*) channels. Scale bars = 100 μm. White arrow show circular colonies. Green arrows displays single cells that disseminated from the nearby colonies and show MT1-MMP protein. Red arrow shows an F-actin protrusion emanating from a circular colony (**c**) Single cells, F-actin disseminations, F-actin protrusions, and zsGreen protrusions were quantified from 20× magnification 3D volumes acquired after MCF-7 MT1-MMP cell lines stably expressing zsGreen were embedded in Matrigel for 5 days. The 60× magnification 3D volume of MT1-MMP C3 cells shows both F-actin (*red arrow*) and zsGreen (*blue arrow*) protrusions emerging from a colony. Scale bars = 100 μm

**Fig. 10:**
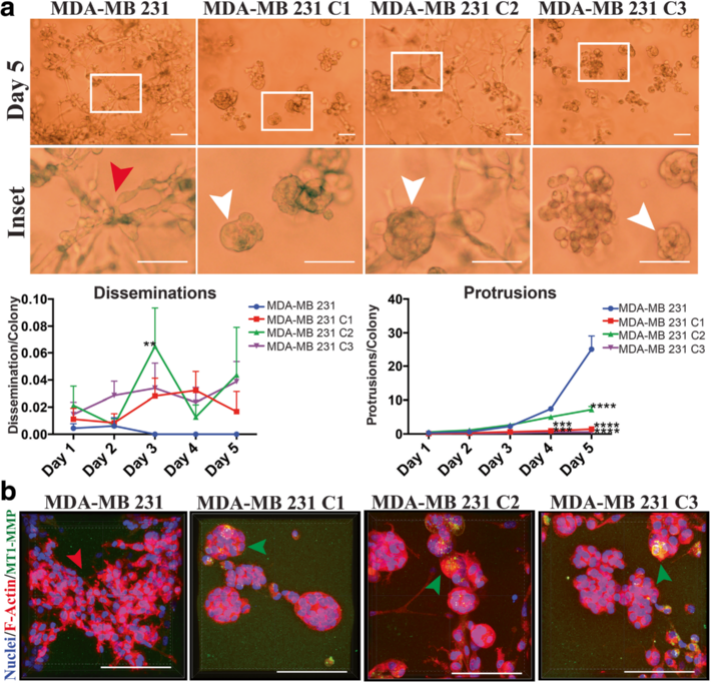
MT1-MMP overexpression inhibited the protrusive morphology of MDA-MB 231 breast cancer cells in 3D culture. **a** (*top*) MDA-MB 231 MT1-MMP cells were embedded in Matrigel and imaged every day for 5 days at 10× magnification. Shown are representative fields of view of each cell line at day 5 and a respective inset. Red arrow shows a portion of the protrusive network MDA-MB 231 cells form in 3D culture. White arrows show MDA-MB 231 MT1-MMP cell colonies that have retained circularity after 5 days in 3D culture. Scale bars = 100 μm. (*bottom*) Five z-stacks per cell line were acquired every day for 5 days and disseminations and protrusions were quantified per colony for each cell line. **b** Representative 3D volume views of immunofluorescence analysis after MDA-MB 231 MT1-MMP cells were embedded in Matrigel for 5 days. Samples were imaged using confocal microscopy at 60× magnification and are displayed as overlays showing MT1-MMP signal (*green*), DAPI (*blue*) and Alexa633 phalloidin (*red*) channels. Scale bars = 100 μm. Red arrow shows protrusive MDA-MB 231 cells, whereas green arrows show circular colonies in MDA-MB 231 MT1-MMP cell lines that are positive for MT1-MMP protein signal. Scale bars = 100 μm

As our work using in vitro 2D culture demonstrated that increased, but relatively low levels of MT1-MMP expression were optimal for enhanced survivability and migration, we subsequently examined how these observations translated to ex vivo 3D culture. In this culture system breast cancer cells are embedded in Matrigel, whose composition resembles the components of a natural BM, and as such mimics a more physiologically relevant 3D environment [43]. MCF-7 MT1-MMP cell lines were embedded in Matrigel and cultured for 5 days in media containing 10 % FBS. During this time, cell morphology was monitored using DIC microscopy to examine how MT1-MMP expression affected cell behavior. Non-invasive MCF-7 cells have been shown to form and remain in circular colonies during 3D culture [50] (Fig. 9a, white arrow), which resemble the acini-like structures that normal breast cells form that are representative of the terminal ductal lobular unit (TDLU) in the human breast [51]. In contrast, invasive MDA-MB 231 cells lose this circular morphology and develop into an interconnected network within the Matrigel (Fig. 10a, red arrow).

During 3D culture, C1, C2 and C3 cells do not form an interconnect network as seen with MDA-MB 231 cells. Instead, their colonies remained circular but display two distinct phenotypes; disseminations - distinct particles being released from circular colonies (Fig. 9a, green arrow), and short dynamic protrusions emerging from colonies (Fig. 9a, red arrows), which were confirmed using time-lapse microscopy (Additional file 8: Figure S3a, Additional files 9, 10, 11 and 12). Comparison of these morphological features between cell lines were blindly quantified per 10× magnification fields of view each day for 5 days and normalized to the number of circular colonies per field of view (Fig. 9a, line graphs). This quantification demonstrated that MCF-7 expressing high levels of MT1-MMP (C1 and C2) had significantly higher number of disseminations per colony than MCF-7 and C3 cells, particularly C1 cells which showed a time-dependent release of particles (Additional file 10). In contrast, the number of protrusions per colony at day 5 was inversely proportional to MT1-MMP expression, as C2, and especially C3 cells had significantly more protrusions per colony, whereas C1 cells had a significantly lower number of protrusions than MCF-7 cells. Immunofluorescence analysis of these MCF-7 MT1-MMP cell lines (Fig. 9b), confirmed that while MCF-7 colonies retained circularity during 3D culture (white arrows), C1 and C2 cells had clear disorganization of colony structure with released cell fragments (disseminations) containing MT1-MMP protein (green arrows). In contrast, C3 cells demonstrated long F-actin protrusions emerging from circular colonies (red arrows), consistent with the protrusive phenotype.


Additional file 9. Time-lapse analysis of 3D culture dynamics of MCF-7 MT1-MMP cells. Parental MCF-7 (Additional file 9), and MT1-MMP C1, C2, and C3 (Additional files 10, 11 and 12, respectively) cells were embedded in 50 % matrigel and incubated in a live imaging chamber at 37 °C, 5 % CO_2_. Z-stacks (100 μm, 5 μm slices) were acquired using a Leica DM16000 B microscope. Frames were taken every 30 min for 72 h and focal planes showing colony features with the greatest clarity were compiled into time-lapse movies using ImageJ. Representative stills of each cell line at three different time points are shown in Additional file 8: Figure S3. Scale bars = 100 μm. (AVI 14794 kb)



Additional file 10. Time-lapse analysis of 3D culture dynamics of MCF-7 MT1-MMP cells. Parental MCF-7 (Additional file 9), and MT1-MMP C1, C2, and C3 (Additional files 10, 11 and 12, respectively) cells were embedded in 50 % matrigel and incubated in a live imaging chamber at 37 °C, 5 % CO_2_. Z-stacks (100 μm, 5 μm slices) were acquired using a Leica DM16000 B microscope. Frames were taken every 30 min for 72 h and focal planes showing colony features with the greatest clarity were compiled into time-lapse movies using ImageJ. Representative stills of each cell line at three different time points are shown in Additional file 8: Figure S3. Scale bars = 100 μm. (AVI 14660 kb)



Additional file 11. Time-lapse analysis of 3D culture dynamics of MCF-7 MT1-MMP cells. Parental MCF-7 (Additional file 9), and MT1-MMP C1, C2, and C3 (Additional files 10, 11 and 12, respectively) cells were embedded in 50 % matrigel and incubated in a live imaging chamber at 37 °C, 5 % CO_2_. Z-stacks (100 μm, 5 μm slices) were acquired using a Leica DM16000 B microscope. Frames were taken every 30 min for 72 h and focal planes showing colony features with the greatest clarity were compiled into time-lapse movies using ImageJ. Representative stills of each cell line at three different time points are shown in Additional file 8: Figure S3. Scale bars = 100 μm. (AVI 14676 kb)



Additional file 12. Time-lapse analysis of 3D culture dynamics of MCF-7 MT1-MMP cells. Parental MCF-7 (Additional file 9), and MT1-MMP C1, C2, and C3 (Additional files 10, 11 and 12, respectively) cells were embedded in 50 % matrigel and incubated in a live imaging chamber at 37 °C, 5 % CO_2_. Z-stacks (100 μm, 5 μm slices) were acquired using a Leica DM16000 B microscope. Frames were taken every 30 min for 72 h and focal planes showing colony features with the greatest clarity were compiled into time-lapse movies using ImageJ. Representative stills of each cell line at three different time points are shown in Additional file 8: Figure S3. Scale bars = 100 μm. (AVI 15106 kb)


To further characterize this novel dissemination phenotype mediated by MT1-MMP, the cellular composition of released fragments was examined by repeating the immunofluorescence analysis with the zsGreen MCF-7 tagged cells. The fluorescently tagged variants were used to provide information about the origin of morphological structures as zsGreen accumulated in the cytoplasm and could be used to better visualize the origins of disseminations or protrusions. The number of single cells (marked by DAPI), F-actin disseminations, F-actin protrusions, and zsGreen protrusions were quantified per colony from 20× magnification 3D volume views (Additional file 8: Figure S3b). This quantification demonstrates that the disseminations are likely enucleated cells, as C1 cells show significantly higher levels of single cells (0.18/colony) and F-actin disseminations (0.67/colony) (Fig. 9c), which added together represented a density of ~0.85/colony, similar to the ~1 dissemination/colony seen by day 5 in our DIC microscopy analysis (Fig. 9a). In contrast, C3 cells demonstrate higher density of F-actin (0.65/colony) and zsGreen protrusions (0.15/colony), which when added together represent a density of ~0.8/colony similar to the ~1 protrusion/colony seen by day 5 in our DIC analysis, confirming that these cells demonstrate a protrusive morphology in 3D culture. Volume views at 60× magnification also showed C3 colonies (Fig. 9c) that demonstrated many zsGreen protrusions (blue arrow) that emerged from existing F-actin protrusions (red arrow). These protrusions are indicative of invadopodia, which are characterized by extension of the cell body due to F-actin polymerization, and subsequent F-actin retraction to result in a protrusion devoid of F-actin [35].

To corroborate our 3D observations with the MCF-7, C1, C2 and C3 cell lines, we performed a similar analysis with MDA-MB 231 MT1-MMP cell lines. MDA-MB 231 cells progressively lost circular morphology and developed into an interconnect network of cells within the Matrigel (Fig. 10a, red arrow), represented by a significantly higher number of protrusions per colony (~25) than MCF-7 cells. In contrast, all MDA-MB 231 cells expressing MT1-MMP showed a significant inhibition to form networks in 3D culture and instead retained a large proportion of circular colonies (Fig. 10a, white arrows). As well, MDA-MB 231 MT1-MMP cells demonstrated a nonsignificant increase in the number of disseminations (~0.4 dissemination/colony), although it was comparatively lower than in MCF-7 MT1-MMP cells. Immunofluorescence analysis of F-actin and nuclear structures in 3D culture confirmed these observations and showed that parental MDA-MB 231 cells formed an extensive cellular network, whereas MDA-MB 231 MT1-MMP cells retained circular colonies marked by MT1-MMP protein (Fig. 10b, green arrows). Taken together, this ex vivo analysis demonstrated that low MT1-MMP expression mediated a protrusive phenotype in 3D culture, shown by C3 (Fig. 9a) and MDA-MB 231 cells (Fig. 10a), whereas high levels of MT1-MMP expression (C1, C2, and MDA-MB 231 MT1-MMP cells) inhibited the ability to form protrusive networks in 3D culture and instead resulted in abnormal release of cell fragments.

### Low levels of MT1-MMP expression mediated a tumorigenic metastatic phenotype in vivo

**Fig. 11:**
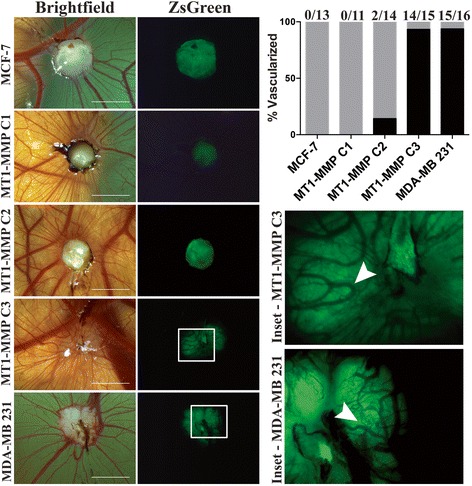
MCF-7 MT1-MMP C3 cells displayed high tumorigenic potential when implanted onto the avian embryo CAM. MCF-7 MT1-MMP cell lines and MDA-MB 231 cells stably expressing zsGreen were implanted into the CAM of day 9 *ex ovo* chicken embryos and visualized 8 days post –implantation using a fluorescence stereoscope to analyze tumor vascularization. Displayed are representative bright field images showing the area of implantation on the embryo, and respective fluorescent images showing the zsGreen channel. The white boxes outline the insets showing vascularization of the MT1-MMP C3 and MDA-MB 231 tumours. Bar graph shows percentage of tumours that were vascularized (*N* ≥ 11). Scale bars = 2 mm

We then tested the in vivo implications of our in vitro observations by using the *ex ovo* chicken embryo [52] and zsGreen expressing MCF-7 C1, C2, and C3 cell lines. These cells, along with zsGreen MDA-MB 231 cells, were suspended in Matrigel and implanted into the ChorioAllantoic Membrane (CAM) of day 9 *ex ovo* chicken embryos [45]. Eight days post implantation the resulting xenograft was visualized using a fluorescent stereoscope to assess vascularization of the formed tumour. No MCF-7 or C1 tumours were scored as vascularized, whereas a minority (2/14) of C2 tumours demonstrated vascularization by vessels of the chicken embryo (Fig. 11). In contrast, the majority of C3 (14/15) and MDA-MB 231 (15/16) tumours were vascularized, shown by the presence of non-fluorescent vessels present within the zsGreen tumour (Additional file 13: Figure S4, white arrow).

**Fig. 12:**
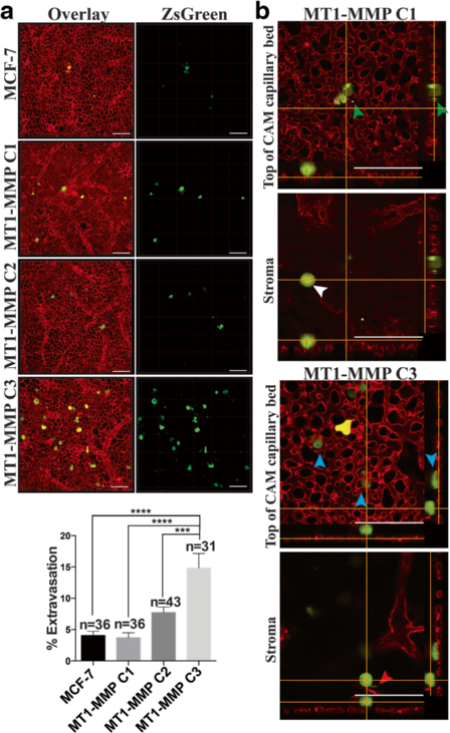
MT1-MMP expression was inversely correlated to the extravasation efficiency of MCF-7 breast cancer cells in vivo. **a** Representative 3D volume views at 20× magnification of MCF-7 MT1-MMP cells stably expressing zsGreen 24 h-post intravenous injection into the chicken embryo CAM vasculature. Shown is an overlay displaying the zsGreen cells (*green*) and CAM vasculature and underlying stromal vessels labeled using lectin-rhodamine (*red*), and the isolated zsGreen channel. Scale bars = 100 μm. Bar graph shows quantification of extravasation efficiency of MCF-7 MT1-MMP cell lines 24 h post-injection. **b** Orthogonal views of Z-stacks acquired using confocal microscopy at 60× of MT1-MMP C1 and C3 cells 24 h post-injection showing the top of the CAM capillary bed (*top*) and the underlying stroma (*bottom*). Extravasated MT1-MMP C1 cells display loss of cell fragments (*green arrows*) and membrane blebbing (*white arrow*), whereas MT1-MMP C3 cells extravasate to below the CAM with uniform morphology (*blue arrows*) and are capable of forming invasive protrusions in the stroma (*red arrow*). Scale bars = 100 μm


The metastatic potential of these cell lines was subsequently examined by direct intravenous injection of zsGreen MCF-7 C1, C2, and C3 cell lines into the vasculature of day 14 chicken embryos, followed by assessment of extravasation efficiency 24 h post-injection [37]. Extravasation out of the vasculature is a necessary step that precedes metastatic colony formation and as such has been shown to directly correlate with metastatic potential [4]. MCF-7 and C1 cells, which express the highest level of MT1-MMP, demonstrate poor extravasation efficiency, as < 5 % of cells were able to extravasate after 24 h (Fig. 12a). C2 cells showed a higher but non-significant increase in percentage of extravasated cells (~8 %), whereas C3 cells, which express low levels of MT1-MMP, displayed a significant increase in extravasation efficiency (~15 %) compared to all other MCF-7 cell lines. Orthogonal sections of extravasated C1 and C3 cells acquired using confocal microscopy at 60× magnification showed that C1 cells are capable of extravasating out of the CAM vasculature (Fig. 12b) but display membrane blebbing (white arrow) and cell fragment release (green arrow), reminiscent of our observations in 3D culture (Fig. 9). In contrast, C3 cells exhibited a uniform morphology as they extravasated into the stromal space (blue arrows) and contained cell protrusions trailing from the CAM capillary bed into the stroma (Additional file 14, red arrow), suggesting that these cells formed invadopodia in vivo*.*

Additional file 14. Real-time intravital imaging of extravasated MCF-7 MT1-MMP C3 cells. MCF-7 MT1-MMP C3 cells stably expressing zsGreen (green) were injected intravenously into the CAM vasculature of day 13 chicken embryos and imaged 24 h post-injection after labeling the vessels with lectin rhodamine (red). A movie in real-time was acquired using the resonant scanner of a Nikon A1R+ confocal microscope. The focus of the microscope was moved manually to show that a single C3 cells has extravasated from the CAM vasculature and shows a protrusion into the stroma (devoid of lectin-rhodamine signal). Scale bar = 100 μm. (MOV 9808 kb)


### Metastatic 21 T breast epithelial cell line produces undetectable levels of MT1-MMP protein

**Fig. 13:**
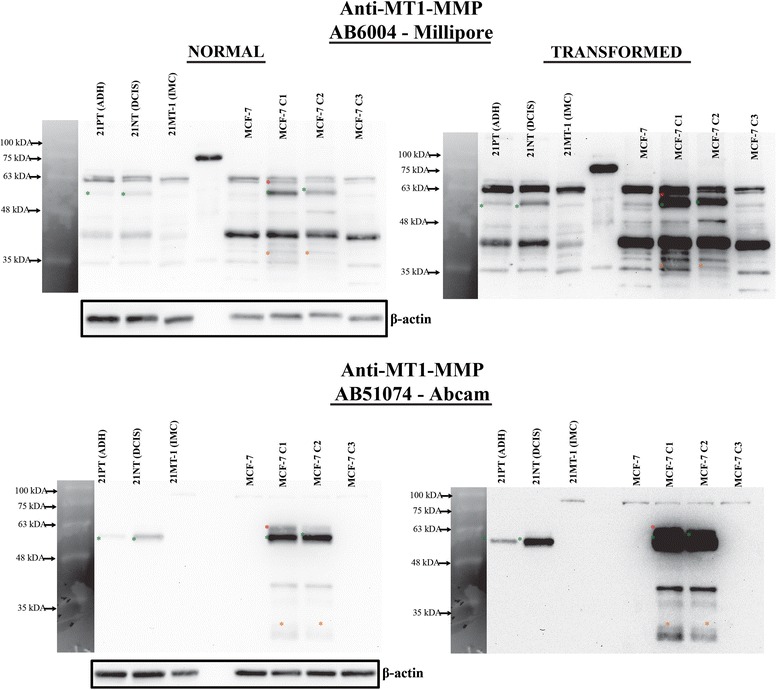
Metastatic human 21 T breast cancer cells showed undetectable levels of MT1-MMP protein similar to MCF-7 C3 cells. Protein lysate from human 21 T breast cancer cell lines, which represent a progression series from atypical ductal hyperplasia (21PT-ADH), to ductal carcinoma in situ (21NT – DCIS), to invasive mammary carcinoma (21MT-1- IMC), were analyzed via immunoblot for MT1-MMP protein levels along with the MCF-7 MT1-MMP cell lines. The blots were probed with either AB6004 (*top*) or AB51074 (*bottom*) and shown as the normal exposure and as transformed versions to clearly show banding pattern. Asterisks indicate MT1-MMP isoforms (green – pro form, red- active form, orange – degradation forms). β-actin was used as a loading control

**Fig. 14:**
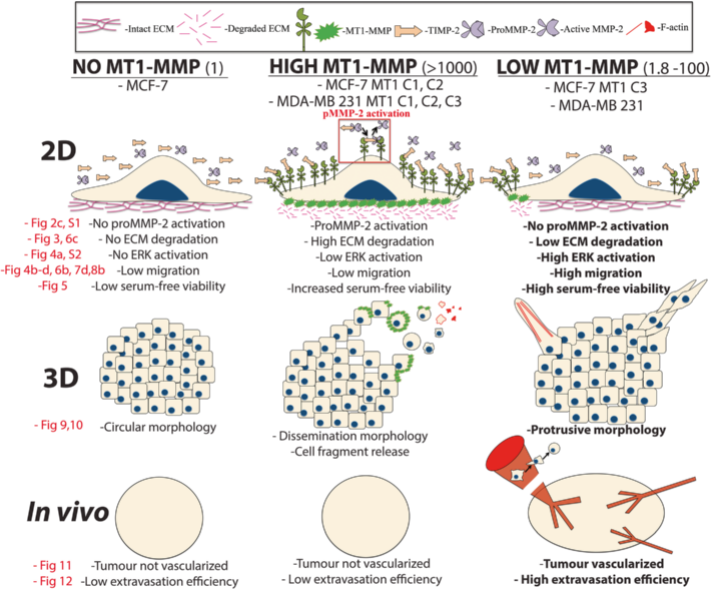
Schematic overview of MT1-MMP expression levels and associated changes in substrate degradation and cell migration in 2D culture, phenotypes in 3D culture, and tumourigenesis in vivo. Schematic representation of the findings of this study showing cell phenotypes across 2D and 3D culture platforms and in vivo. Legend describing molecular components in diagrams is shown at the top, and fold change relative to MCF-7 parental cells is in the brackets to the right of the bolded titles. MT1-MMP deficient breast cancer cells, such as MCF-7 cells, are incapable of proMMP-2 activation or ECM degradation, and show low migration and viability during serum-free incubation. These cells retain a circular morphology in 3D culture, and do not form vascularized tumours nor display high extravasation efficiency in vivo. Cells expressing high levels of MT1-MMP are capable of proMMP-2 activation and widespread ECM degradation, have increased survivability to serum-free stress, but do not demonstrate increased migration in 2D experiments. In 3D culture, these cells demonstrate a dissemination morphology and cell fragment release mediated by MT1-MMP. Despite MT1-MMP protein production and associated substrate degradation, these cells are unable to form vascularized tumours or increase their extravasation efficiency in vivo. Cells expressing low levels of MT1-MMP do not demonstrate proMMP-2 activation or widespread ECM degradation, but do show increased migratory potential, and high viability during serum-free incubation. These cells demonstrate a protrusive morphology in 3D culture, form vascularized tumours in vivo, and have significantly increased extravasation efficiency. Data figures within this study that correspond to the diagrams within this model are in red text

To extend and corroborate our observations that low levels of MT1-MMP are optimal to promote metastatic features in 3D culture and in vivo, we assayed via immunoblot the level of MT1-MMP protein in the 21 T series cell lines. These cells were isolated from a single patient and represent a mammary tumor progression series that mimic specific stages of breast cancer progression [39, 53] (atypical ductal hyperplasia -21PT-ADH, ductal carcinoma in situ -21NT – DCIS and invasive mammary carcinoma- 21MT-1- IMC) (Fig. 13). Assaying MT1-MMP protein levels in these cell lines demonstrated that ADH and DCIS variants produced active MT1-MMP, with the non-invasive DCIS cells producing higher levels of MT1-MMP protein. Direct comparison to the MCF-7 MT1-MMP cell lines showed that C1 and C2 cells produced more active MT1-MMP than ADH or DCIS 21 T cells. In contrast, 21MT-1 cells, which represent an invasive mammary carcinoma, produced undetectable levels of MT1-MMP protein as determined by immunoblot, similar to C3 cells. The 21MT-1 IMC cells have been show to possess metastatic qualities in 3D culture and in vivo when compared to the non-invasive variants [39]*,* consistent with our observations that low levels of MT1-MMP are representative of metastatic breast cancer. Taken together, the observations in our study demonstrate that low levels of MT1-MMP expression are optimal for tumorigenicity and metastatic potential in vivo*,* and importantly, that abnormally high MT1-MMP overexpression correlated with a decrease, rather than enhancement of tumorigenic features (see schematic representation Fig. 14).

## Discussion

In this study, we utilized overexpression of functional MT1-MMP in MCF-7 and MDA-MB 231 breast cancer cells and demonstrated how overexpression corresponds to proMMP-2 activation and ECM degradation, but inversely correlates to migratory potential, protrusive phenotype in 3D culture, and tumorigenic features in vivo. Instead we showed that high overexpression of MT1-MMP negatively affects cell viability, and causes an abnormal loss of colony structure and cell fragment release in 3D culture that translates to decreased tumorigenic potential in vivo. We also demonstrated using the human 21 T cell lines mammary tumour progression series that breast cancer cells which mimic an invasive mammary carcinoma (IMC) are better represented by low, rather than high, levels of MT1-MMP protein. Our data is at odds with the notion that high MT1-MMP expression is crucial for tumour progression, as numerous studies report that MT1-MMP overexpression is associated with enhanced migratory ability and tumourigenecity [17, 26, 27, 33, 34, 54], although there is also evidence in agreement with our study which shows that high MT1-MMP overexpression is insufficient to increase metastasis of human cancer cells [31]. Additionally, despite the mounting evidence demonstrating that MMPs have a direct role in basement membrane degradation in vitro*,* there is no evidence to date that shows this is the case in vivo*,* and instead such studies suggest that the role of MMPs may not be related to ECM degradation but rather proteolysis of non-ECM substrates (Reviewed in [9]).

Here, using MCF-7 clonal cell lines stably expressing untagged MT1-MMP, we show that migration, as shown by a scratch closure assay and by time-lapse microscopy of cells on fluorescent substrate, is dependent on levels of MT1-MMP, with high levels decreasing migratory ability and low levels promoting it. We also demonstrate that migration changes seen using transwells involve a substantial cell viability component, where high MT1-MMP overexpression negatively affects viability and low levels enhances it. This viability difference likely contributed to the magnitude of migration augmentation between transwell and other assays. Studies that show an increase in cancer cell migration as a result of MT1-MMP overexpression demonstrate migration enhancement ranging from 50 -500 % [26–28], whereas others show a requirement of TIMP-2 for MT1-MMP mediated migration enhancement [24, 25]. These reports do not agree on a specific mechanism, except general ERK activation. In contrast, there are studies demonstrating that MT1-MMP overexpression does not increase migration of breast cancer cells [55], and also that MT1-MMP overexpression decreases ERK activation in cancer cells [56]. In the latter study, the authors demonstrated that MT1-MMP overexpression in various cancer cell lines, including MCF-7 cells, downregulates ERK activation and migration in response to FGF-2, which is consistent with our findings using cells that express high levels of MT1-MMP. Other studies have provided strong mechanistic evidence that MT1-MMP is involved in apoptosis protection [21] and viability enhancement via HIF1α stabilization [23, 57], which are in line with our observations that MT1-MMP enhances viability during serum-free incubation.

A striking finding of our in vitro analysis regarding the relationship between MT1-MMP expression and migration was that the most migratory cells were the ones which had a low MT1-MMP:high TIMP-2 ratio. Of the MCF-7 MT1-MMP cell lines used, C3 and C3 SH 1 cells displayed low MT1-MMP levels (11 and 1.8 fold change vs parental MCF-7 cells, respectively), and their migration ability was greatly enhanced when the levels of TIMP-2 increased, especially C3 SH 1 cells. MDA-MB 231 MT1-MMP cell lines displayed the same trend whereby MT1-MMP overexpression with no change in TIMP-2 expression (data not shown) shifted the ratio in favor of excess MT1-MMP, and thereby causing a decrease in migratory potential. Similarly, analysis of the natural migration potential of MCF-7, MDA-MB 231, and HS578t cells is consistent with this relationship to TIMP-2, as HS578t cells were the most migratory and displayed the highest level of TIMP-2 relative to MT1-MMP. Noteworthy in this analysis was also the observation that MDA-MB 231 cells displayed the highest ERK activation, but were not the most migratory. This was consistent with our comparison of MCF-7 C2 and C3 cells, whereby C2 cells showed the highest ERK activation but were less migratory than C3 cells, which displayed comparatively lower ERK activation even in the presence of high levels of TIMP-2.

Although TIMP-2 is a natural MMP inhibitor and as such has attracted therapeutic interest along with synthetic MMP inhibitors [10, 58–62], neither have shown value in clinical trials [63]. Instead, some have suggested that high TIMP-2 levels may promote tumourigenecity [24, 64, 65], which has been strengthened by the association of high TIMP-2 levels with poor prognosis in various human cancers, including breast [66–71]. Although this association between an MMP inhibitor and poor cancer prognosis may be paradoxical, we describe here that while TIMP-2 regulation of MT1-MMP activity is complex, as exemplified by MDA-MB 231 and HS578t cells, high TIMP-2:low MT1-MMP ratios in these cells correlate with their migratory potential, and also with their lack of gelatin degradation and inhibited proMMP activation ability. Despite the fact that MDA-MB 231 and HS578t cells naturally express MT1-MMP, MMP-2, and −9, the extracellular gelatinases are predominately found in their pro-forms yet to be activated. As MT1-MMP activity is pivotal in gelatinase activation [20], this indicates that MT1-MMP present in these cells is inhibited, likely by TIMP-2. This is consistent with lack of gelatinase activity of C3 cells, and in stark contrast to C1 and C2 cells, suggesting that cells with non-physiologically high MT1-MMP exhibit excessive proteolysis which may be counterproductive to migration and cell viability. This is corroborated both with the rescued serum free viability and migration of C1 cells as a result of BB94 treatment, and with the role of TIMP-2 in mediating survivability under serum free conditions as shown by others [21]. Since MT1-MMP is a proteolytic enzyme that can cleave and alter the function of many ECM and non-ECM proteins crucial for proper cell behavior [18], it is logical that such a potent protease with wide substrate specificity would be under tight control by TIMP-2 to appropriately mediate cell behaviour.

Maden and Bugge (2015) analyzed the last two decades of literature to examine if there was a consensus regarding the cellular source of MMPs (including MT1-MMP) in human cancers and whether they were predominately stromal or cancer cell derived. These authors noted that publications were widely inconsistent in regards to the cellular source of MMPs, particularly when immunodetection was involved. Only when in situ hybridization was used was there a consensus seen that MMPs were likely stromal cell derived [29]. The authors proposed reasons for these difficulties, one being that there is likely inherently low expression of MMPs in cancer cells compared to stromal cells making immunodetection technically challenging.

We believe that unreliable immunodetection reagents (discussed in [72]) is a major contributing reason as to why there is such inconsistency when assessing the abundance of MMPs in human cancers and their value as prognostic markers. In our study, we initially experienced difficulties assessing immunoblots for MT1-MMP, which could only be correctly interpreted after examining the immunological banding pattern for MT1-MMP expressing MCF-7 and MDAMB-231 cell lines and probing with two different primary antibodies against MT1-MMP (Additional file 15: Figure S5). To strengthen the idea that improper immunodetection of MT1-MMP protein can lead to incorrect conclusions, we highlight our (lack of) immunodetection of MT1-MMP in MCF-7 breast cancer cells. We strongly believe, as suggested by others, that MCF-7 cells are MT1-MMP deficient [24, 33], particularly because it has been shown that the MT1-MMP promoter in these cells is hypermethylated and thus transcriptionally repressed [73]. Yet despite this observation, published studies claim to detect both pro- and active MT1-MMP protein via immunoblot in these cells [34, 74], which could be due to incorrect identification of the cell line used for experimentation, or lack of stringency when conducting immunodetection. As can be seen from our immunoblot data, usage of a polyclonal antibody against MT1-MMP resulted in a non-specific signal that could easily be misinterpreted as pro- and active MT1-MMP in MCF-7 cells. Furthermore, in the study done by Köhrmann et al., although the authors reported MT1-MMP protein in MCF-7 cells, they were not able to detect MT1-MMP protein from clinical samples via immunoblot. However, they were able to detect MT1-MMP protein in tissue sections from tumour samples and not from normal tissues. Studies that are internally inconsistent regarding MT1-MMP protein detection, and which contain clinical samples, can create confounding conclusions regarding the role of MMPs in cancer and are in agreement with the observations of Madsen and Bugge regarding the discrepancies of the source of MMPs in human cancer. Additionally, visualizing MT1-MMP protein at a cellular level using immunofluorescence may also lead to similar immunodetection problems. Lodillinsky et al. recently implicated the p63/MT1-MMP axis in the transition from in situ to metastatic breast cancer, reporting that MT1-MMP protein is present during BM invasion of MCF10DCIS.com xenografts. However, with the knowledge that MT1-MMP is localized to distinct specialized regions of the cell membrane to initiate invasion (invadopodia), we question such immunofluorescence data that show MT1-MMP protein is present throughout the cell membrane of every cell in the xenograft, regardless of whether it is in physical proximity to invade the BM.

To assess the physiological relevance of observed levels of MT1-MMP expression in this study, we compared these to recently reported MT1-MMP mRNA levels in malignant and non-malignant human breast tissues. The reported increase in MT1-MMP mRNA levels between malignant tissues and normal tissues ranged between ~1.7 and ~3.2 fold [75–77]. Similarly, a pioneering study used MDA-MB 231 variants that produced constitutively active scr kinase, which is known to be upregulated during cancer progression [35]. These MDA-MB 231 variants generated significantly more MT1-MMP containing invadopodia. Analysis of MT1-MMP mRNA changes between control and constitutively active src kinase cells demonstrated a ~1.8 fold change increase in MT1-MMP mRNA, which the authors describe as a mechanistically meaningful increase in MT1-MMP expression. Therefore, the physiological relevance of extreme changes in expression levels, such as our ~17,000 fold change in MT1-MMP mRNA seen in our transient transfectants, or ~1500 fold change in stable cell lines, would be difficult to reconcile with primary human breast cancers which have a ~1.7 to 3.2 fold change in MT1-MMP mRNA expression. Additionally, in line with our idea that immunological reagents of MT1-MMP may be unreliable, if normal non-malignant tissues do not contain detectable levels of MT1-MMP [17, 34] and cancerous tissues demonstrate only a ~1.7 to 3.2 fold increase in MT1-MMP mRNA, then is it reasonable that a transcriptional increase of that magnitude would be difficult to immunodetect at the protein level.

Consistent with the conclusion of the importance of low levels of MT1-MMP expression are the observations seen in our physiologically relevant ex vivo and in vivo experiments, whereby MCF-7 cells expressing low levels of MT1-MMP (C3) demonstrated behavior consistent with the role of MT1-MMP during cancer progression. C3 cells have increased protrusive morphology in 3D culture and this is contrasted with MCF-7 cells overexpressing high levels of MT1-MMP which show reduced protrusive ability and increased cell fragmentation. Similarly, C3 cells were tumorigenic and showed metastatic potential in vivo, unlike C1 and C2 cells, which is consistent with studies that knock down MT1-MMP expression and show inhibition of these parameters. Additionally, analysis of MT1-MMP protein levels in the 21 T cell lines mammary tumour progression series demonstrated that breast cancer cells which represent ADH and DCIS mammary tumours produce high levels of active MT1-MMP protein, whereas invasive 21MT-1 IMC cells produce undetectable levels of MT1-MMP, an observation that is consistent with our findings using MCF-7 C3 cells and our overall conclusion that low levels of MT1-MMP may better represent metastatic cancer. A similar study using the HMT-3522 epithelial cell series yielded results consistent with our analysis of MT1-MMP levels in 21 T cells, as these authors analyzed microarray data to show that MMP-9, −13,-15 and −17, but not MT1-MMP, were functionally significant in the acquisition of invasiveness [78].

Interestingly, the observation that DCIS 21 T cells produced high levels of active MT1-MMP in comparison to their IMC counterparts is similar to the 3D culture phenotype of our MDA-MB 231 cells overexpressing MT1-MMP. In our study, parental MDA-MB 231 cells, which are naturally invasive, readily form irregular networks in 3D culture in contrast to non-invasive cell lines that partially maintain polarity and form acini similar to the TDLU in the human breast (eg MCF-7 cells). It was surprising to us to that overexpression of MT1-MMP in invasive MDA-MB 231 cells reverted their phenotype in 3D culture towards a DCIS-like morphology where the ability to form networks in matrigel 3D culture was restricted and a higher proportion of these cells retain acini-like colonies. The reversion of MDA-MB 231 cells to a DCIS-like phenotype in 3D culture as a result of MT1-MMP overexpression is consistent with our analysis of MT1-MMP protein levels in the 21 T cell lines whereby DCIS (21NT) cells produce more active MT1-MMP and predominately form acini in 3D culture, and IMC (21MT-1) cells produce less MT1-MMP protein and display invasive 3D behavior [39], similar to our MT1-MMP MDA-MB 231 cells and parental MDA-MB 231 cells, respectively.

Taken together, this study shows that a physiologically relevant increase in MT1-MMP expression is at best represented by a 1.8 to 11-fold change compared to normal tissue. Additionally, although abnormally high levels of MT1-MMP overexpression may not reflect those seen in primary breast cancers, there is still mechanistic value in this approach, as utilized in this study to demonstrate the constancy of the TIMP-2 mediated activation of proMMP-2 by MT1-MMP. With this work we want to challenge the long-standing view that MMPs, particularly MT1-MMP, exert their role in cancer progression as proteases that predominately degrade ECM components to allow cancer cell invasion, and instead suggest a subtle role for MT1-MMP in tumour progression as metastatic cancer appears to be better represented by low levels of inhibited MT1-MMP protein.

## Conclusions

Our findings that low levels of MT1-MMP are physiologically relevant suggest that very high levels MT1-MMP overexpression represents a non-relevant level of MT1-MMP expression. Excessive ECM degradation mediated by high levels of MT1-MMP is not permissive to cell migration and tumourigenesis, while low levels of MT1-MMP promote extravasation and vascularization in vivo.

## Additional files

Additional file 1: Figure S1. MCF-7 breast cancer cells expressing high levels of MT1-MMP displayed the well-characterized relationship of TIMP-2 mediated proMMP-2 activation. (a) MCF-7 cells were transiently transfected with TIMP-2 or ALA + TIMP-2 and the protein lysate and media were collected for analysis using immunoblot (top) and reverse zymography (bottom) to examine TIMP-2 protein levels. β-actin was used as a loading control. Reverse zymography analysis shows that TIMP-2 inhibits MMP activity (dark bands), whereas ALA + TIMP-2 is devoid of this function (light bands) due to the non-functional N-terminal domain. (b) Reverse zymography of MCF-7 and MT1-MMP cell lines incubated for 15 min or 12 h with TIMP-2 or ALA + TIMP-2 CM diluted 1:100 to show TIMP-2 levels in the media post-incubation. Serum free media with CM diluted from mock-transfected cells was used as a control. (c) Immunoblot analysis of serially diluted recombinant TIMP-2 and TIMP-2 CM. The image of the bottom blot is a transformed version of the top blot to visualize the banding pattern for the recombinant TIMP-2 samples. Based on densitometry analysis (not shown), the concentration of TIMP-2 protein in TIMP-2 CM is estimated to be approximately 10 μg/ml. (d) MCF-7 and MT1-MMP cell lines were incubated for 12 h with proMMP-2 CM supplemented with increasing dilutions of TIMP-2 or ALA + TIMP-2 CM (1:100; 1 part CM, 100 parts SF media) and then this media was assayed using zymography and reverse zymography to assess proMMP-2 activation and TIMP-2 levels, respectively. Low levels (1:100) of TIMP-2 enhance activation of proMMP-2 by MCF-7 cells expressing high levels of MT1-MMP (red arrows), whereas high levels of TIMP-2 (Full CM;undiluted) inhibit this activation process (blue arrows). (JPG 2995 kb)
Additional file 2: Figure S2. MCF-7 breast cancer cells expressing low levels of MT1-MMP and exposed to high levels of TIMP-2 showed rapid activation of the ERK pathway. Representative immunoblot analysis of ERK1/2 activation after MCF-7 and MT1-MMP expressing cell lines were seeded and incubated for 12 h (top) or 15 min (bottom) with media containing 10 % FBS or SF media supplemented with different dilutions of TIMP-2 or ALA + TIMP-2 CM (CM:SF). Total ERK1/2 was used as a loading control. Quantification of these immunoblots is shown in Fig. 4a. (JPG 2168 kb)
Additional file 8: Figure S3. MCF-7 cells expressing high levels of MT1-MMP released cell fragments in 3D culture, whereas cells expressing low levels of MT1-MMP displayed a protrusive migratory phenotype. (a) MCF-7 and MT1-MMP cell lines were embedded in 50 % Matrigel and incubated for 5 days in media containing 10 % FBS. Between days 2 and 5, samples were placed in a live imaging chamber and imaged at 20× magnification at the same stage position every 30 min for 72 h to make timelapse movies showing cell dynamics in 3D culture (Additional files 9, 10, 11 and 12). Shown are stills at three different time points for each sample showing colonies that retain circularity (white arrows), release cell fragments (green arrows) or display protrusions and migratory behavior (red arrows). Scale bars = 100 μm. (b) MCF-7 and MT1-MMP cell lines stably expressing zsGreen were embedded in 50 % Matrigel for 5 days and processed for immunofluorescence to visualize nuclei, zsGreen protein, MT1-MMP protein and F-actin distribution. Samples were imaged using confocal microscopy at 20× magnification and displayed as a 3D volume overlay and the individual channels. Green squares show single cells surrounding a colony, white squares highlight the insets on the right showing F-actin disseminations (green arrows) and F-actin protrusions (red arrows). Quantification of this analysis is shown in Fig. 9c. Scale bars = 100 μm. (JPG 5795 kb)
Additional file 13: Figure S4. Excision of vascularized tumours demonstrated internal blood vessels. A tumour from MDA-MB 231 cells expressing zsGreen was excised from a chicken embryo 8 days post-implantation and imaged using brightfield and fluorescence microscopy. Vessels within the tumour (white arrows) can be seen by the presence of blood (brightfield) and absence of fluorescent signal (zsGreen), which indicate that these vessels originate from the chicken embryo. Cutting this tumour in half and rotating the pieces to reveal the inside of the tumour confirms internal vascularization. Scale bars = 2 mm. (JPG 6034 kb)
Additional file 15: Figure S5. Immunological detection of MT1-MMP protein can be confounding depending on antibody utilized. (a) Immunoblot analysis of MCF-7 and MDA-MB 231 MT1-MMP cell lines (top), or MCF-7, MDA-MB 231 and HS578t breast cancer cell lines (bottom) using a polyclonal anti-MT1-MMP rabbit antibody (AB6004 – Millipore). (b) Immunoblot analysis of MCF-7, MDA-MB 231, and HS578t breast cancer cells, along with the MCF-7 MT1-MMP cell lines, using a monoclonal rabbit antibody (AB51075- Abcam). These blots were ran for 6 h at 140 V on a 15 % acrylamide gel to ensure optimal band separation. On the left is a normal exposure of each blot and on the left is transformed version to clearly demonstrate banding pattern. Arrows indicate non-specific signal, whereas asterisks indicate specific signal pertaining to MT1-MMP isoforms (green – pro form, red- active form, orange – degradation forms). Note the substantial amount of non-specific signal obtained when using AB6004 compared to AB51074, despite both antibodies being able to specifically detect multiple isoforms of MT1-MMP. Of particular interest are the red and green non-specific bands obtained using AB6004, which could be misinterpreted as pro- and active forms of MT1-MMP, respectively (see banding pattern for MT1-MMP deficient MCF-7 cells). (JPG 3520 kb)

## Abbreviations

BM: Basement membrane
CAM: ChorioAllantoic membrane
CD-44: Cluster of differentiation 44
CM: Conditioned media
DIC: Differential interference contrast
ECM: Extracellular matrix
ERK: Extracellular regulated kinase
F-actin: Filamentous actin
HIF: Hypoxia inducible factor
MMPs: Matrix metalloproteinases
MT1-MMP: Membrane type 1 matrix metalloproteinase
PH3: Phospho-histone 3
SF: Serum free
TIMP-2: Tissue inhibitor of metalloproteinases 2
