# Supplementary figures and images for: Less is more: low expression of MT1-MMP is optimal to promote migration and tumourigenesis of breast cancer cells
Source: Mol Cancer. 2016 Oct 18;15:65. doi: 10.1186/s12943-016-0547-x (PMC5070195; doi:10.1186/s12943-016-0547-x)

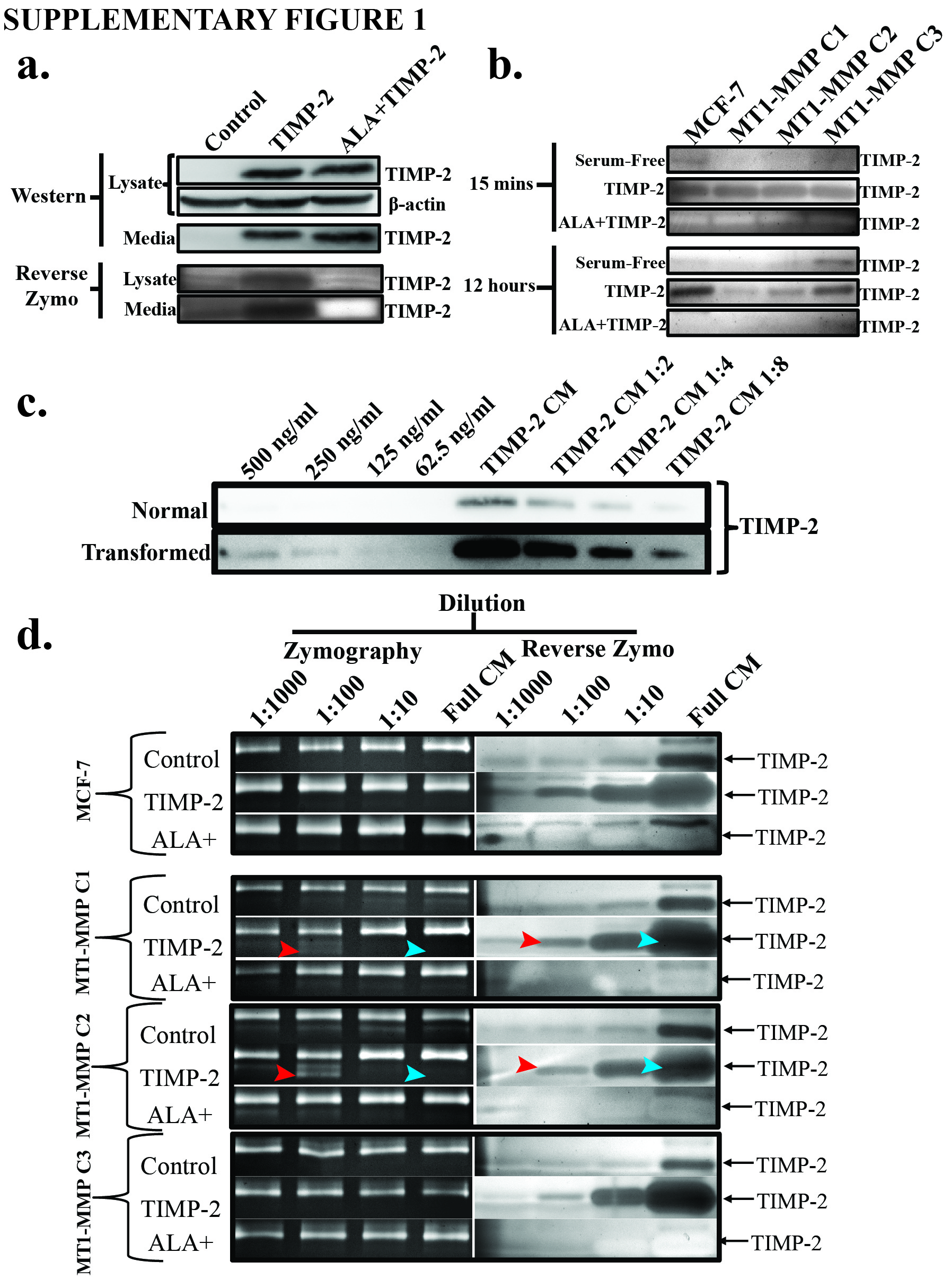

Supplement: Additional file 1: Figure S1. — MCF-7 breast cancer cells expressing high levels of MT1-MMP displayed the well-characterized relationship of TIMP-2 mediated proMMP-2 activation. (a) MCF-7 cells were transiently transfected with TIMP-2 or ALA + TIMP-2 and the protein lysate and media were collected for analysis using immunoblot (top) and reverse zymography (bottom) to examine TIMP-2 protein levels. β-actin was used as a loading control. Reverse zymography analysis shows that TIMP-2 inhibits MMP activity (dark bands), whereas ALA + TIMP-2 is devoid of this function (light bands) due to the non-functional N-terminal domain. (b) Reverse zymography of MCF-7 and MT1-MMP cell lines incubated for 15 min or 12 h with TIMP-2 or ALA + TIMP-2 CM diluted 1:100 to show TIMP-2 levels in the media post-incubation. Serum free media with CM diluted from mock-transfected cells was used as a control. (c) Immunoblot analysis of serially diluted recombinant TIMP-2 and TIMP-2 CM. The image of the bottom blot is a transformed version of the top blot to visualize the banding pattern for the recombinant TIMP-2 samples. Based on densitometry analysis (not shown), the concentration of TIMP-2 protein in TIMP-2 CM is estimated to be approximately 10 μg/ml. (d) MCF-7 and MT1-MMP cell lines were incubated for 12 h with proMMP-2 CM supplemented with increasing dilutions of TIMP-2 or ALA + TIMP-2 CM (1:100; 1 part CM, 100 parts SF media) and then this media was assayed using zymography and reverse zymography to assess proMMP-2 activation and TIMP-2 levels, respectively. Low levels (1:100) of TIMP-2 enhance activation of proMMP-2 by MCF-7 cells expressing high levels of MT1-MMP (red arrows), whereas high levels of TIMP-2 (Full CM;undiluted) inhibit this activation process (blue arrows). (JPG 2995 kb) [file 12943_2016_547_MOESM1_ESM.jpg]

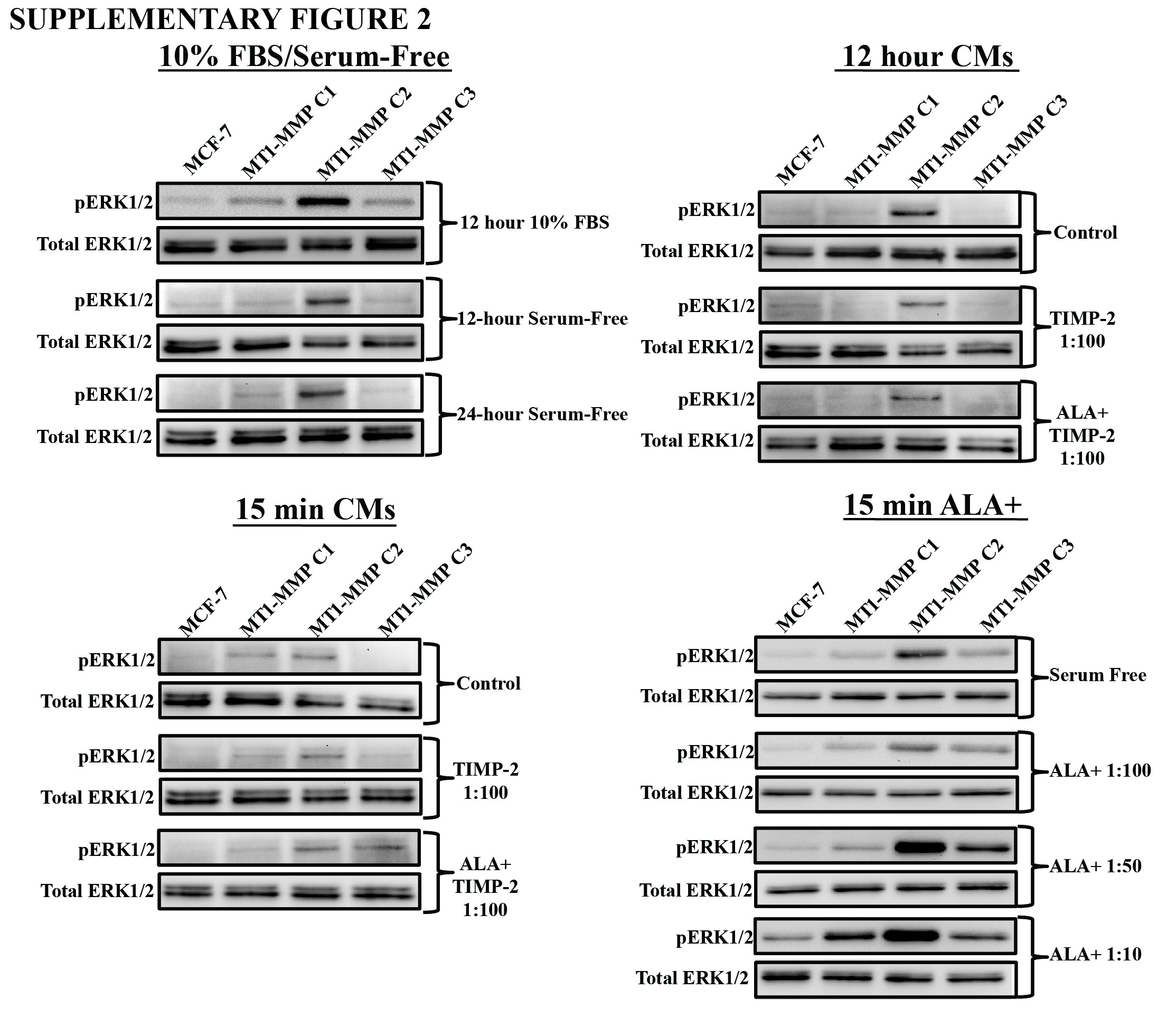

Supplement: Additional file 2: Figure S2. — MCF-7 breast cancer cells expressing low levels of MT1-MMP and exposed to high levels of TIMP-2 showed rapid activation of the ERK pathway. Representative immunoblot analysis of ERK1/2 activation after MCF-7 and MT1-MMP expressing cell lines were seeded and incubated for 12 h (top) or 15 min (bottom) with media containing 10 % FBS or SF media supplemented with different dilutions of TIMP-2 or ALA + TIMP-2 CM (CM:SF). Total ERK1/2 was used as a loading control. Quantification of these immunoblots is shown in Fig. 4a. (JPG 2168 kb) [file 12943_2016_547_MOESM2_ESM.jpg]

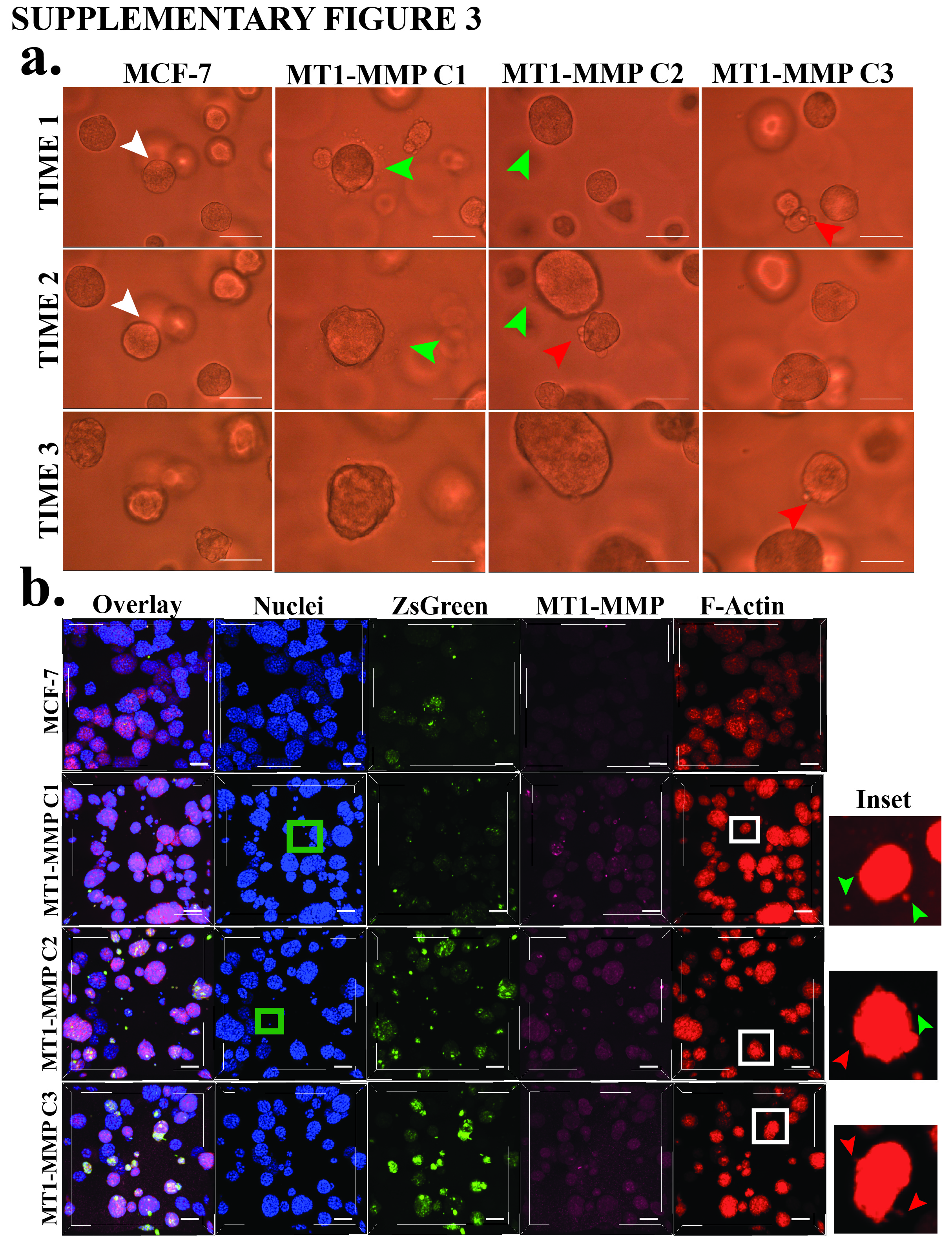

Supplement: Additional file 8: Figure S3. — MCF-7 cells expressing high levels of MT1-MMP released cell fragments in 3D culture, whereas cells expressing low levels of MT1-MMP displayed a protrusive migratory phenotype. (a) MCF-7 and MT1-MMP cell lines were embedded in 50 % Matrigel and incubated for 5 days in media containing 10 % FBS. Between days 2 and 5, samples were placed in a live imaging chamber and imaged at 20× magnification at the same stage position every 30 min for 72 h to make timelapse movies showing cell dynamics in 3D culture (Additional files 9, 10, 11 and 12). Shown are stills at three different time points for each sample showing colonies that retain circularity (white arrows), release cell fragments (green arrows) or display protrusions and migratory behavior (red arrows). Scale bars = 100 μm. (b) MCF-7 and MT1-MMP cell lines stably expressing zsGreen were embedded in 50 % Matrigel for 5 days and processed for immunofluorescence to visualize nuclei, zsGreen protein, MT1-MMP protein and F-actin distribution. Samples were imaged using confocal microscopy at 20× magnification and displayed as a 3D volume overlay and the individual channels. Green squares show single cells surrounding a colony, white squares highlight the insets on the right showing F-actin disseminations (green arrows) and F-actin protrusions (red arrows). Quantification of this analysis is shown in Fig. 9c. Scale bars = 100 μm. (JPG 5795 kb) [file 12943_2016_547_MOESM8_ESM.jpg]

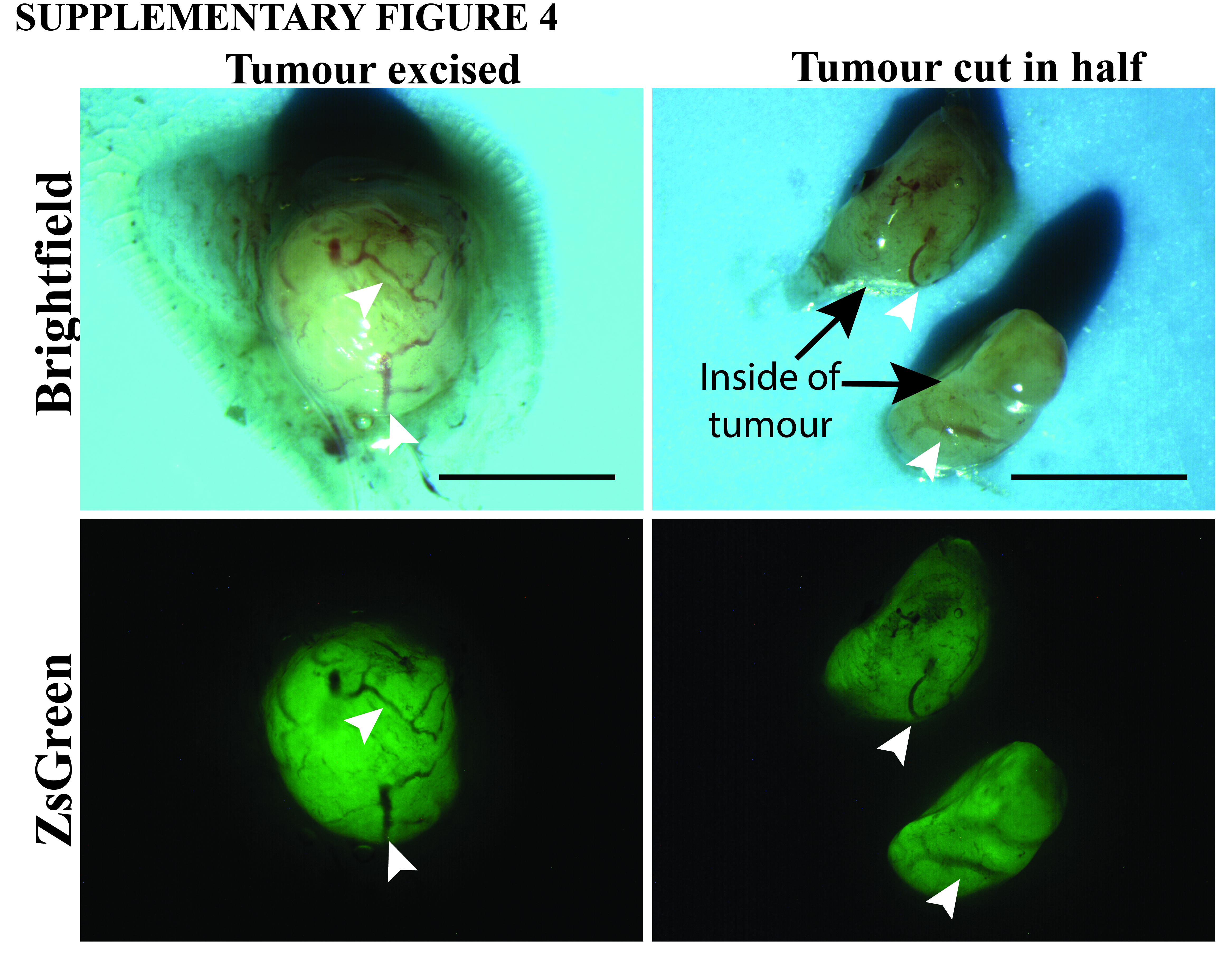

Supplement: Additional file 13: Figure S4. — Excision of vascularized tumours demonstrated internal blood vessels. A tumour from MDA-MB 231 cells expressing zsGreen was excised from a chicken embryo 8 days post-implantation and imaged using brightfield and fluorescence microscopy. Vessels within the tumour (white arrows) can be seen by the presence of blood (brightfield) and absence of fluorescent signal (zsGreen), which indicate that these vessels originate from the chicken embryo. Cutting this tumour in half and rotating the pieces to reveal the inside of the tumour confirms internal vascularization. Scale bars = 2 mm. (JPG 6034 kb) [file 12943_2016_547_MOESM13_ESM.jpg]

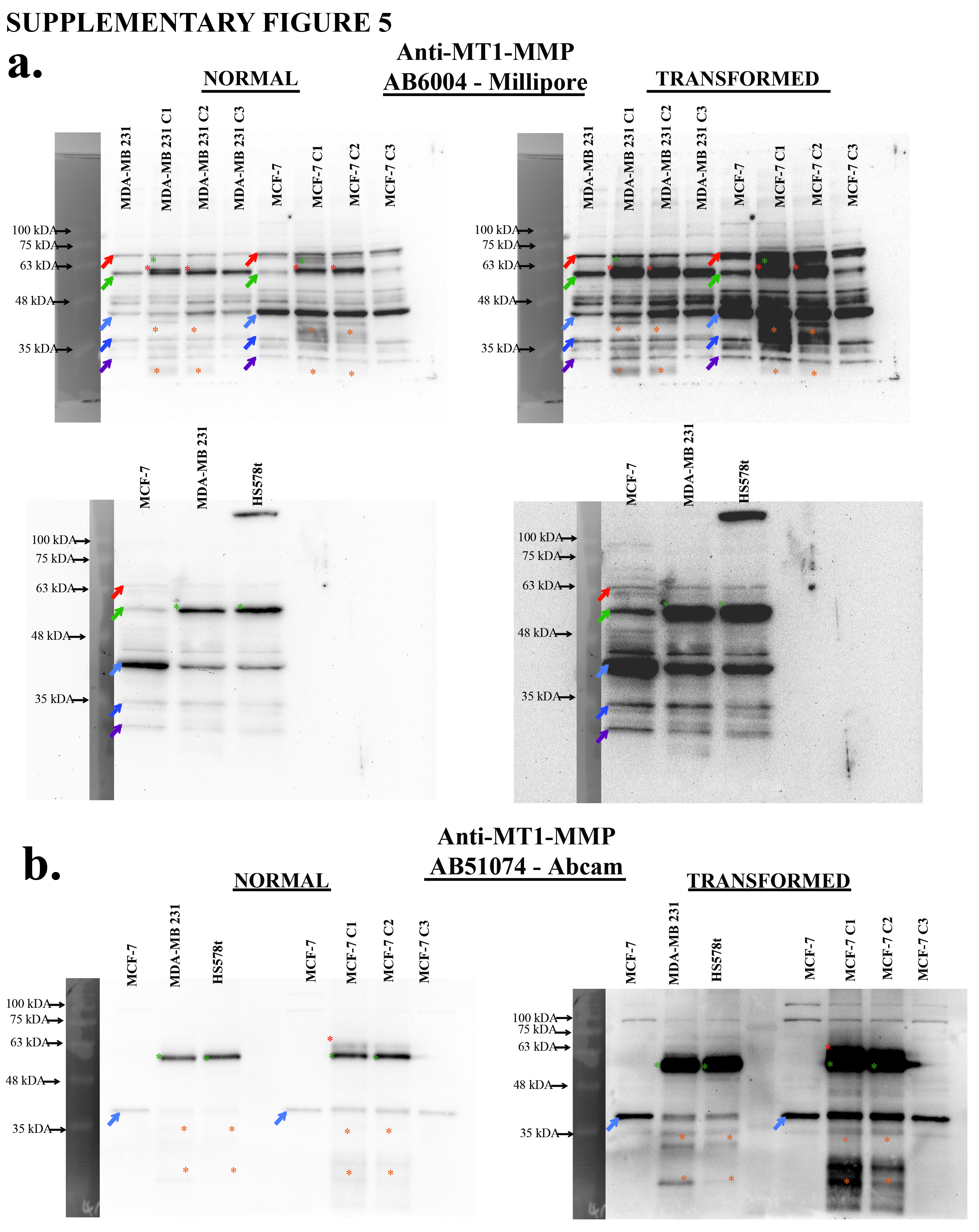

Supplement: Additional file 15: Figure S5. — Immunological detection of MT1-MMP protein can be confounding depending on antibody utilized. (a) Immunoblot analysis of MCF-7 and MDA-MB 231 MT1-MMP cell lines (top), or MCF-7, MDA-MB 231 and HS578t breast cancer cell lines (bottom) using a polyclonal anti-MT1-MMP rabbit antibody (AB6004 – Millipore). (b) Immunoblot analysis of MCF-7, MDA-MB 231, and HS578t breast cancer cells, along with the MCF-7 MT1-MMP cell lines, using a monoclonal rabbit antibody (AB51075- Abcam). These blots were ran for 6 h at 140 V on a 15 % acrylamide gel to ensure optimal band separation. On the left is a normal exposure of each blot and on the left is transformed version to clearly demonstrate banding pattern. Arrows indicate non-specific signal, whereas asterisks indicate specific signal pertaining to MT1-MMP isoforms (green – pro form, red- active form, orange – degradation forms). Note the substantial amount of non-specific signal obtained when using AB6004 compared to AB51074, despite both antibodies being able to specifically detect multiple isoforms of MT1-MMP. Of particular interest are the red and green non-specific bands obtained using AB6004, which could be misinterpreted as pro- and active forms of MT1-MMP, respectively (see banding pattern for MT1-MMP deficient MCF-7 cells). (JPG 3520 kb) [file 12943_2016_547_MOESM15_ESM.jpg]
